# Opposition to voluntary and mandated COVID-19 vaccination as a dynamic process: Evidence and policy implications of changing beliefs

**DOI:** 10.1073/pnas.2118721119

**Published:** 2022-03-22

**Authors:** Katrin Schmelz, Samuel Bowles

**Affiliations:** ^a^Cluster of Excellence “The Politics of Inequality,” University of Konstanz, 78457 Konstanz, Germany;; ^b^Thurgau Institute of Economics, 8280 Kreuzlingen, Switzerland;; ^c^Santa Fe Institute, Santa Fe, NM 87501

**Keywords:** public health policy compliance, crowding out intrinsic motivation, trust, cognitive dissonance, control aversion

## Abstract

COVID-19 vaccination rates slowed in many countries during the second half of 2021, along with the emergence of vocal opposition, particularly to mandated vaccinations. Who are those resisting vaccination? Under what conditions do they change their minds? Our three-wave representative panel survey from Germany allows us to estimate the dynamics of vaccine opposition, providing the following answers. Without mandates, it may be difficult to reach and to sustain the near-universal level of repeated vaccinations apparently required to contain the Delta, Omicron, and likely subsequent variants. But mandates substantially increase opposition to vaccination. We find that few were opposed to voluntary vaccination in all three waves of the survey. They are just 3.3% of our panel, a number that we demonstrate is unlikely to be the result of response error. In contrast, the fraction consistently opposed to enforced vaccinations is 16.5%. Under both policies, those consistently opposed and those switching from opposition to supporting vaccination are sociodemographically virtually indistinguishable from other Germans. Thus, the mechanisms accounting for the dynamics of vaccine attitudes may apply generally across societal groups. What differentiates them from others are their beliefs about vaccination effectiveness, their trust in public institutions, and whether they perceive enforced vaccination as a restriction on their freedom. We find that changing these beliefs is both possible and necessary to increase vaccine willingness, even in the case of mandates. An inference is that well-designed policies of persuasion and enforcement will be complementary, not alternatives.

## Significance

The challenge of securing adherence to public health policies is compounded when an emerging threat and a set of unprecedented remedies are not fully understood among the general public. The evolution of citizens’ attitudes toward vaccination during the COVID-19 pandemic offers psychologically and sociologically grounded insights that enrich the conventional incentives- and constraints-based approach to policy design. We thus contribute to a behavioral science of policy compliance during public health emergencies of the kind that we may increasingly face in the future. From early in the pandemic, we have tracked the same individuals, providing a lens into the conditions under which people’s attitudes toward voluntary and mandated vaccinations change, providing essential information for COVID-19 policy not available from cross-section data.

The challenge of the first half-year of most COVID-19 vaccination campaigns was the supply of vaccines, not demand for them. It then appeared that, once vaccines were widely available, vaccination rates would approach the two-thirds level initially thought to be sufficient to control the pandemic. But the Delta and then Omicron variants along with substantial and persistent reported opposition to vaccination have raised doubts about whether vaccine willingness will be sufficient. In response, by the end of 2021, vaccination mandates were being widely adopted by governments, businesses, and educational institutions, and universal mandates were being considered and implemented (1–8).

The appropriate strategies for raising vaccination rates depend on the target rate, on how many are unlikely to be vaccinated willingly in a sufficiently timely manner, on the conditions under which opponents change their minds, and on the effect of the policies themselves on vaccination preferences. Our study sheds light on the behavioral side of this evaluation.

## Three-Wave Panel Survey on Attitudes toward Voluntary and Mandated Vaccinations

Our online panel survey was designed to allow insights into the dynamics of opposition to COVID-19 vaccination as well as the evolution of responses to voluntary versus enforced vaccination policies over the pandemic. Each of the three cross-sections making up the panel survey (conducted in April–May 2020, October–November 2020, and May 2021; *SI Appendix*, Fig. S2) is composed of about 4,000 respondents that are representative of the German population. Our panel consists of the 2,018 individuals who participated in all three waves. The panel is very similar to the three representative cross-sections in the respondents’ sociodemographics and attitudes toward vaccinations (as shown in *SI Appendix*, Table S2). The German government’s explicit endorsement of a voluntary-only vaccination regime did not change over the course of the three waves of the survey. Only in late 2021 was a transition to greater use of mandates initiated (see the timeline of developments over the course of the pandemic in *SI Appendix*).

Vaccinations began late in Germany; just 6.7% of our May 2021 survey respondents had been vaccinated twice (considered at that time as “fully vaccinated”). Those who were not yet vaccinated twice were asked: “To what extent would you agree with being vaccinated yourself if: … vaccination is strongly recommended by the government but remains voluntary? … vaccination is made mandatory and controlled by the government?” Answers were given on a five-point Likert scale ranging from zero (“not agree at all”) to four (“fully agree”).

We asked about agreement with being vaccinated rather than intended compliance with a mandate were one to be imposed so as not to confound our phenomenon of interest—the respondent’s attitude, that is “being okay with vaccination”—with their behavior, abiding with a legal requirement (see *Materials and Methods* for further explanations).

## Results

Our survey responses and the subsequent trajectory of vaccinations provide a measure of the validity of the survey, albeit an imperfect one. Access to the vaccine was restricted to priority groups until June. How accurate would have been a naïve prediction based on our May survey? The prediction would be that, once the vaccine became available to all adults, by late July, the numbers vaccinated would approach that of those who, in May 2021, agreed with being vaccinated, and that, from then onward, the rate of vaccinations would substantially decline because the remaining unvaccinated were more hesitant.

According to Our World in Data (9), 61% of the total German population was vaccinated at least once by late July, which corresponds to 73% of the adult population (as shown in *SI Appendix*). This is in line with the 73% of those we surveyed in May who strongly or weakly favored vaccination if voluntary (Likert response levels 3 or 4) or already had been vaccinated twice. Vaccination rates declined substantially after reaching that share of the population, as a naïve prediction based on the survey would have anticipated [from a rate of growth of 1.37% daily between mid-May and late July to 0.16% daily thereafter until 18 November, when the German government announced that health sector workers would be required to be vaccinated (10)] (*SI Appendix*, Fig. S3).

### Will Mandates Crowd Out Vaccination Willingness?

A possible adverse effect of this shift from voluntary to mandatory vaccinations—crowding out people’s intrinsic willingness to be vaccinated—was an important concern early in 2021 when most of the unvaccinated in Germany, the United States, and other countries were not opposed to being vaccinated. Our survey evidence from the first two waves of the pandemic in Germany (11, 12) showed that making vaccination a legal requirement might have retarded the rate of vaccinations, as it would have substantially reduced willingness to be vaccinated, consistent with self-determination and reactance theory in psychology and what economists term “control aversion” (13–18). This crowding-out effect is confirmed in our third-wave evidence (*SI Appendix*, Fig. S4).

**Fig. 1.**
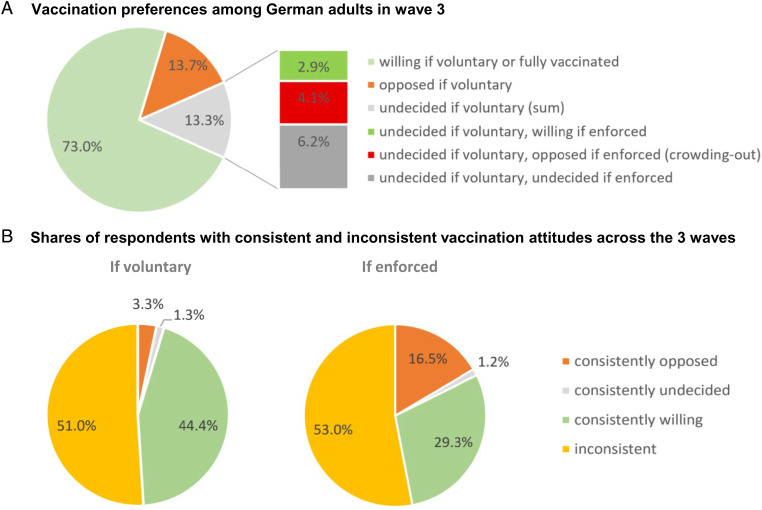
(*A*) Vaccination preferences among German adults in wave 3. (*B*) Shares of those consistently opposed (i.e., choosing levels 0 or 1 in each of the three survey waves), consistently willing, consistently undecided, and inconsistent across the three waves. In *A*, the pie captures the shares under the voluntary vaccination regime being in place at the time of the survey, and the bars indicate the responses of those who were undecided under voluntary vaccination policies were enforced vaccination to be implemented. The 73% making up the green slice of the pie are composed of those expressing agreement with being vaccinated voluntarily (levels 3 and 4) plus those vaccinated twice (at that time considered as “fully vaccinated”). Among the 13.7% opposed if voluntary, 9.8% would remain opposed if enforced, 1.1% would be undecided, and 2.8% would become willing (not shown in the figure); *n* = 4,021 answered those questions in cross-section wave 3. In *B*, three-waves panel: *n* = 2,018.

What would have been the effect on the attitudes of the unvaccinated were a vaccine mandate to have been imposed in late July, when the fraction vaccinated reached 73%, the naïve prediction? If, hypothetically, the remaining unvaccinated were distributed as were the opposed or undecided for the case of voluntary vaccinations in our May 2021 survey, constituting 13.7% and 13.3%, respectively, of those surveyed, then the distribution would be as illustrated by the pie chart in Fig. 1*A*.

The figure shows that a vaccine mandate would have had little net effect on the extent of pro or con attitudes. Roughly half of the undecided would have remained undecided, our survey suggests (the dark gray bar in Fig. 1*A*), with the other half split between those who would have become opposed (the red bar) numbering only slightly more than those who would have responded to a mandate by switching to favoring vaccinations (the green bar).

Thus, in contrast to earlier in 2021, the cost of a mandate in terms of crowding out of intrinsic motivation and increased opposition among the remaining unvaccinated and undecided would have been small. However, the declining efficacy of the vaccines and the enduring pandemic means that policy makers have to be concerned about those who quickly got vaccinated twice, among whom there may be an adverse effect of mandates for ongoing vaccine willingness. 

### The Dynamic Nature of Vaccination Willingness: How Stable Is Opposition?

Exploiting the panel nature of our data, we find that opposition to vaccination is far from immutable. While, in every wave, a substantial fraction opposes vaccination, Fig. 1*B* shows that only a small minority (3.3% of our panel) consistently opposed voluntary vaccination over all three waves (that is, less than a fifth of the 16.9% who expressed opposition to voluntary vaccination at a given point in time, averaging over the three cross-sections). In contrast, for the case of mandated vaccinations, consistent opposition was expressed by 16.5% (half of the 33.2% of opposition to enforced vaccination averaged over the cross-sections). Three-quarters of the consistently opposed 16.5% in the case of mandated vaccination had expressed willingness to be vaccinated voluntarily in at least one survey wave, suggesting that mandating vaccinations early in the pandemic could have hardened what was then a transient opposition.

While fluid, opposition to vaccination is also very widespread, particularly if mandated: 34.6% were opposed to voluntary vaccination in at least one of the survey waves, and 52.4% were opposed if vaccination were mandated. We observe a downward trend in opposition to voluntary vaccinations (from 20.0% and 18.3% in May and October–November 2020 to 12.4% in May 2021 in the panel), but not for enforced vaccination (29.3%, 42.6%, and 31.6% in the panel). Opposition to voluntary COVID-19 vaccinations in our May 2021 survey (12.4%) was thus only modestly greater than opposition to the familiar and well understood measles vaccination when it was still voluntary in Germany (7.6%) (19).

**Fig. 2.**
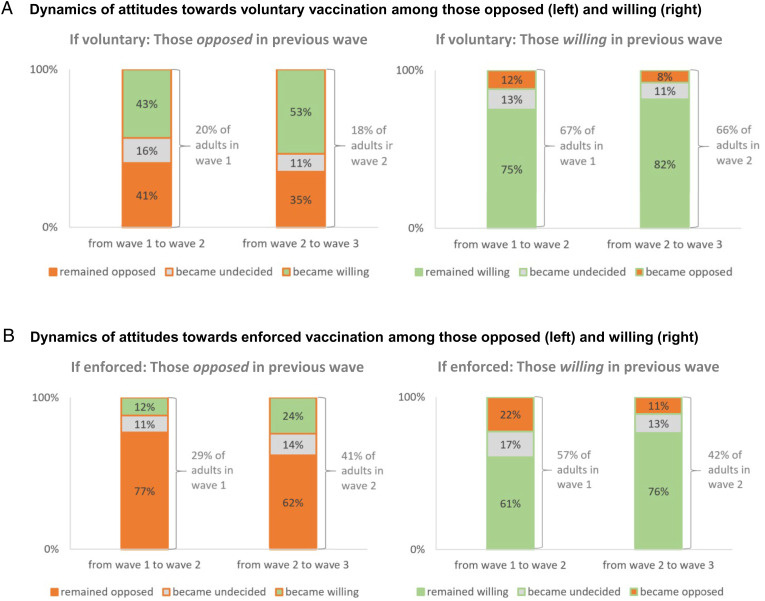
For the cases of voluntary (*A*) and enforced (*B*) vaccinations, how those opposed (*Left*) or willing (*Right*) in one wave of the survey responded in the next wave. In each chart, the segments of the bars show responses in the subsequent survey wave of those who had been opposed to vaccination in wave 1 (first bar) and wave 2 (second bar). For example, the top segment of the left bar in *A, Left* shows that 43% of those opposed to voluntary vaccination in wave 1 (20% of the adult population indicating level 0 or 1) were willing to get vaccinated voluntarily in wave 2 (indicating agreement level 3 or 4); *n* = 2,542 answered those questions in waves 1 and 2, and *n* = 1,903 in waves 2 and 3 (full three-waves panel excluding those vaccinated twice in wave 3).

Fig. 2 illustrates the transience of opposition to vaccination. In the case of voluntary vaccination, many of those opposed in one wave turn to favoring vaccination in the next wave (Fig. 2 *A*, *Left*). Among the 18% who were opposed to voluntary vaccination in wave 2, for example, more than half became willing in wave 3. One-third of those who had been strongly opposed to voluntary vaccination in our second wave (October–November 2020) were already vaccinated at least once at the time of our third wave or had an appointment to be vaccinated (note that, in May 2021, vaccination was still restricted to priority groups). In contrast to the opposed and the undecided (as detailed in *SI Appendix*, Fig. S5), only a small share of the willing changed their minds (Fig. 2, *Right*).

Opposition to enforced vaccinations is more stable: The majority of those opposed in one wave remain opposed in the next (Fig. 2 *B*, *Left*). Of the 41% opposed in wave 2, less than a quarter became willing in wave 3.

We can model the dynamics of vaccination readiness as a Markov process and use the above attitude switching data, expressed as transition probabilities, to derive the stationary (long-run equilibrium) distribution of the population across the five Likert responses. The stationary distribution gives the fractions of the population in each response category (from strongly disagreeing with being vaccinated to strongly agreeing) such that those leaving the category (having changed their minds) are just offset by those arriving (having previously responded some other way, and then changed their minds.) Intuitively, with many switching from opposition to willingness and few switching in the other direction, the stationary fraction of the population in opposition to voluntary vaccinations will necessarily be small, and this is what we find.

We show that, if the transition probabilities between the first and second waves were to have been sustained, the stationary fraction of the population opposed to mandated vaccination would have been 56.4%, while those agreeing with vaccination would have been half that number (*SI Appendix*, Table S7*B*). However, the transition probabilities estimated from the second and third waves imply a stationary distribution almost exactly in reverse (24.9% opposed and 60.4% agreeing; *SI Appendix*, Table S7*D*).

What we observed, therefore, over this period is a substantial reduction of the fraction of those opposed to mandated vaccination that would persist in the long run were the transition probabilities hypothetically to remain constant. Similarly, in the case of voluntary vaccinations, the stationary share of opposition fell from 18%, using waves 1 and 2, to 10%, using waves 2 and 3 (*SI Appendix*, Table S7 *A* and *E*).

The stationary distribution, however, is a thought experiment and not a prediction, because the transition probabilities on which it is based are themselves in flux. For example, the change in the stationary distribution estimated from waves 1 and 2 and from waves 2 and 3 resulting in the stationary share of voluntary vaccination willingness rising (*SI Appendix*, Table S7 *A* and *E*) suggests that the vaccination dynamics could be changing so that those agreeing in the stationary distribution would be substantially greater as of this writing.

### Who Are the Opponents of Vaccination, and What May Change Their Minds?

For the design and targeting of effective vaccination policy, it is important to know whether there are sociodemographic characteristics or particular beliefs that distinguish the consistent opponents from others. To explore this question, we estimate logit equations for both voluntary and mandated vaccinations predicting consistent opposition across the three survey waves.

Our explanatory variables include gender, education, migrant background, childhood in East Germany, and other standard sociodemographic indicators, as well as attitudinal and belief measures such as trust in public institutions (the average of general trust in the federal government, in the state government, in science, and in media), belief in effectiveness of the vaccine, and beliefs about COVID-19 and COVID-19 policies as well as a standard psychological measure of conformism (20) as shown in *SI Appendix*, Table S4 and Figs. S10 and S11.

We find, first, that the sociodemographic characteristics are uninformative in predicting either consistent opposition or moving out of opposition to willingness. We use Tjur’s R^2^, a measure of goodness of fit for a binary dependent variable (21), to determine the relative explanatory power of beliefs and attitudes above and beyond sociodemographic characteristics. The binary dependent variable equals one if the respondent is consistently opposed and equals zero otherwise. Tjur’s R^2^ is the difference in the predicted mean of the dependent variable between those who are consistently opposed and others. This measure thus gives us an intuitive sense of the degree to which our equations capture the true unit difference between the two groups’ vaccination attitudes, including and excluding the measures of beliefs and attitudes. (Tjur’s R^2^ is exactly the fraction of variance explained in the linear probability model.)

In the full model including beliefs and attitudes, the mean of the predicted dependent variable for those consistently opposed to voluntary vaccinations (the ones) is 0.18 and for all others (the zeros) is 0.03. The difference between these two predictions (0.18 − 0.03 = 0.15 = Tjur’s R^2^) means that 15% of the true unit difference between the two sets of respondents is explained by the model.

By contrast, sociodemographic variables alone fail to distinguish between the consistent opponents and the others: Tjur’s goodness of fit measure in this case is 0.007, implying that sociodemographic differences explain less than 1% of the true difference between the two types. In the case of enforced vaccines, the full model including beliefs and attitudes explains 48% of the difference, while the sociodemographic characteristics alone explain 3%. The more commonly used (but less straightforward to interpret) Pseudo R^2^ is qualitatively in line with Tjur’s R^2^, as shown in *SI Appendix*, Table S5.

We also predict moving out of opposition to vaccination between waves 2 and 3 of the survey (October–November 2020 to May 2021; our first wave does not include the full set of beliefs questions). Considering those who were opposed in wave 2, the binary dependent variable equals one if a respondent moves from opposition in wave 2 to willingness in wave 3 and equals zero otherwise. For the case of moving from opposition to voluntary vaccination to willingness, Tjur’s R^2^ for the full model is 35.4%, and Tjur’s R2 for the model including only sociodemographic characteristics is 7.3%. Similar results hold for the case of enforcement, where the Tjur’s R^2^ are, respectively, 36.4% and 5.3%.

The evidence on Tjur’s R^2^ in the regressions with only sociodemographic predictors means that the sociodemographic differences—operating both directly and indirectly through their statistical association with particular beliefs and attitudes—do not meaningfully distinguish those consistently opposed and those moving from opposition to willingness. It appears that, while sociodemographic characteristics predict the level of vaccine willingness in our own and others’ cross-section data (11, 12, 22), the mechanisms accounting for change and stability in vaccine attitudes may apply generally across sociodemographic groups.

**Fig. 3.**
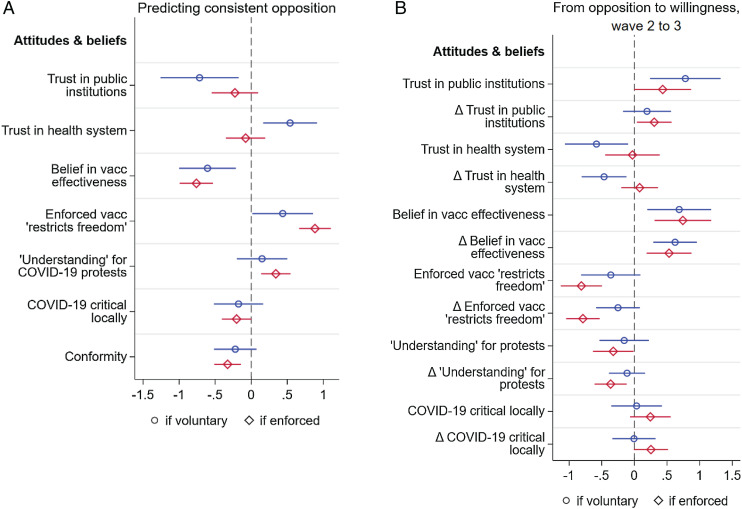
Predicting persistent opposition to vaccination (vacc) in all three survey waves (*A*) and movement from opposition to favoring vaccinations between waves 2 and 3 (*B*) for the cases of voluntary (blue) and mandated (red) vaccinations. Shown are the coefficients and 95% CI, estimated in logistic regressions with standardized variables including sociodemographic measures (the full regressions are provided in *SI Appendix*, Figs. S10 and S11). The *x* axes are in units of standardized coefficient estimates. The first entry of *A* and *B* shows, for example, that trust in public institutions predicts less likelihood of consistent opposition (*A*) and greater likelihood of switching from opposition to willingness (*B*). In *A*, *n* = 1,959, that is, the entire sample. In *B*, the observations are those opposed in wave 2 (*n* = 325 for the voluntary case and *n* = 741 for the mandated case). Estimates of the analogous linear probability models shown in *SI Appendix* are qualitatively similar.

Consistent opposition is associated with the beliefs and attitudes shown in Fig. 3*A*: lack of trust in public institutions, beliefs that the vaccines are ineffective, and trust in the German health system (presumably to take care of the respondent should they become ill). The belief in vaccine effectiveness may reflect trust in scientists (with which it is correlated in our dataset), the importance of which has also been found for other COVID-19 policies (23).

Those who express “understanding for” the demonstrations against COVID-19 policies are more likely to be consistently opposed, and this is particularly the case if vaccination were to be mandated. Plausibly, the more respondents perceive the pandemic situation in their local area as critical, the less likely they are to consistently oppose vaccination. Our measure of conformism is associated with not being a consistent opponent of vaccinations (recall that almost three-quarters in our third wave survey favor vaccinations). Those who perceive enforced vaccinations as a restriction on their “freedom” are more likely to be consistently opposed.

Fig. 3*B* shows that both levels of beliefs and changes in beliefs predict moving from opposition to willingness, especially the belief (and its change) in vaccine effectiveness, the perception that a mandate does not restrict “freedom,” as well as the level and change in trust in public institutions.

To get some idea of the effect size of changes in beliefs or attitudes, we use the easily interpretable coefficients of the linear probability model. For example, an SD increase from wave 2 to wave 3 in one’s belief in vaccine effectiveness is associated with a 12-percentage-point increase from wave 2 to wave 3 in the likelihood of moving from opposition to favoring voluntary vaccination (*SI Appendix*, Fig. S11). This is one-third of the difference predicted by the entire model—the full equation with sociodemographics and beliefs included.

### Our Results Are Unlikely to Reflect Response Error or Unrepresentativeness of the Panel.

Survey responses are, of course, subject to error arising from both recording mistakes and limited self-knowledge (24), the latter of which may be particularly the case in unfamiliar domains such as acceptance of a novel vaccine during an unprecedented pandemic. Could the very limited number of people observed to be consistently opposed to voluntary vaccination reflect response error rather than genuine changes in vaccination attitudes? This appears to be unlikely given the much larger shares of consistently willing and, in the case of mandates, consistently opposed (Fig. 1*B*), as well as in light of our accurate prediction of the trajectory of the numbers that would be actually vaccinated in the months following our third survey wave. (We more fully review the internal evidence on survey error in *SI Appendix*.)

Even if, hypothetically, there were to have been no change at all in vaccination attitudes but, instead, substantial randomness in answers, the estimated number consistently opposing vaccinations would still be small. To see this, suppose that, with some probability (*μ*), survey subjects select one of the five items of the Likert scale at random. When a vaccine opponent responds randomly, they will correctly record opposition to vaccination with probability 0.4 (responding zero or one) and being undecided or favoring vaccinations with probability 0.6 (responding two, three, or four). So, in each of the three waves, those truly opposed to vaccinations would respond “opposed” with probability 1 − 0.6*μ*, and they would respond “opposed” consistently in all three waves with probability (1 − 0.6*μ*)^3^. In each wave those not opposed would erroneously respond “opposed” with probability 0.4*μ* and would erroneously respond “opposed” in all three waves with probability (0.4*μ*)^3^.

Two empirical observations from our survey—in the case of voluntary vaccinations, 3.3% of the population are consistently opposed, and 16.9% are opposed on average across the three waves—provide us with two equations from which we can jointly compute the invariant opposed fraction (*x*) and the error rate (*μ*), both defined over the unit interval,

$$\begin{array}{l} x{\left(1-0.6\mu \right)}^{3}+(1-x){\left(0.4\mu \right)}^{3}=0.033\\ x(1-0.6\mu )+(1-x)0.4\mu =0.169\;. \end{array}$$

The first equation requires that the sum of the truly opposed who respond consistently in opposition plus those truly not opposed who erroneously but consistently respond (in all three waves) as if they were opposed must equal the empirically observed 0.033 of the population. The second equation requires that the predicted fraction expressing opposition in a single wave, that is, the sum of those truly opposed responding without error plus those not opposed but who erroneously responded as if they were opposed, must equal the observed average cross-section opposition of 0.169 of the population.

Solving the two equations simultaneously for *x* and *μ*, we find that the true consistently opposed in this scenario *x* = 0.059 and the random error rate *μ* = 0.322. (The share of those consistently opposed in the case of enforced vaccination computed in the same way is far higher, 0.301, along with an estimated error rate of 0.307.) This hypothetical noise-driven scenario is, of course, unrealistic, but it provides an upper bound on the extent of consistent opposition.

Concerning possible biases due to deviations from a representative sample, while dropouts beyond the researcher’s control are unavoidable in any panel, it is unlikely that our results could be driven by these. The sociodemographic criteria used to define and recruit the representative cross-section samples are very similar in our panel and the three representative cross-section samples (*SI Appendix*, Table S2). As a robustness check, we also use sample weights based on the German microcensus to restore full representativeness of the panel and find that using this sample-weighted data has virtually no effect on the results reported here (*SI Appendix*, Table S3).

To address the possibility that our panel may underrepresent opponents of government vaccination policies, we show that, in the panel (as well as the three cross-sections), the share of those who would vote for the right-wing populist party that has opposed government COVID-19 policies (Alternative für Deutschland, AfD) is virtually identical to the shares of AfD voters in public opinion polls on the dates of our three survey waves. Moreover, vaccination attitudes are very similar in the fully representative cross-sections and the panel (*SI Appendix*, Table S2).

### Is Our Evidence from Germany Informative about Other Countries?

Comparable COVID-19 panels from other countries do not exist, to the best of our knowledge, as most available surveys do not compare responses to voluntary versus mandated vaccinations. Moreover, being cross-sections rather than panels in most cases, they are uninformative about transience or consistency of individual attitudes.

We can, however, contrast the third cross-section wave of our survey with the Kaiser Family Foundation (KFF) US survey (22) for a month when the fraction already vaccinated in the United States was similar to that in our May 2021 survey (we asked the identical KFF questions for this purpose). We find that, while somewhat fewer of the unvaccinated Germans were “definitely not” willing to be vaccinated (16% in Germany and 19% in the United States), the distribution of vaccination attitudes is not markedly different between these two countries (*SI Appendix*, Fig. S6).

The fluidity of opposition to vaccination that we observe points to the limits of cross-section studies. Interpreting the large share consistently opposed to voluntary vaccinations in repeated cross-sections in Germany as a substantial “hard core” of consistent opposition would have been mistaken. This same caution might apply to other countries, for example, the United States, where the share of those responding that they would “definitely not” be vaccinated was effectively constant from December 2020 to January 2022—varying between 12% and 16% with no trend (22).

Even without data from a similar survey in other countries, we can provide some clues on the extent to which our results may be applied to people raised in different cultural–institutional settings. To do so, we exploit the fact that the older cohort of Germans was raised in two radically different environments: liberal West Germany and the centrally planned GDR under Communist Party rule. Cultural–institutional differences between East and West Germany date back at least to the 15th century, with authoritarian social relations in the areas east of the Elbe River dominated by the Junker landlord class and with more liberal institutions in the German free cities in the south and throughout most of what came to be West Germany (25–27).

Thus, while sharing a common language and many cultural attributes, the institutional and cultural heritage of older East and West Germans differed in many respects more than the between-country differences observed, for example, among the uniformly liberal democratic and market-based societies of North America and Western Europe. These East–West German differences are apparent in survey responses and experimental behavior concerning such attitudes as preferences for government interventions in private domains (28), the value of autonomy (29), interpersonal trust (30), the degree of self-interest and solidarity with others (31, 32), and control aversion (11, 33).

Were we to find substantially different results for those raised in the East and the West before German reunification, we would conclude that our results are sensitive to the cultural–institutional environment, cautioning against their applicability to other countries. To explore this concern, we created separate subsamples of East and West Germans who came to maturity prior to reunification in 1990. We then use these subsamples to recompute two separate sets of estimates of our main results, namely, the fraction that consistently opposed voluntary and enforced vaccination, and the extent to which being consistently opposed is predicted by sociodemographic characteristics alone and by the full model including beliefs.

The result is that we would draw the same conclusions from the two subsamples of older East and West Germans. Thus, we do not observe East–West differences in persistent opposition to vaccination and moving from opposition to willingness. Accordingly, even though the distribution of attitudes about vaccination differs between East and West Germans (11), their dynamics may be more universal. While hardly a definitive test of the extent to which our results may be generalized, this is consistent with our results being robust to substantial differences in cultural and institutional heritages and socialization (*SI Appendix*, Figs. S7–S9).

Another piece of evidence suggesting some degree of generality for our results using German data is that experiments on the effects of incentives and constraints on behavior suggest commonalities among European and North American populations, for example, with respect to control aversion and cooperation [although to varying degrees (18, 34–37)]. Moreover, our finding that sociodemographics do not explain the dynamics of opposition to vaccination, those opposing vaccination being better distinguished by their beliefs and attitudes, is consistent with evidence from Ireland and the United Kingdom (38). In the January 2022 KFF survey in the United States, by far the largest difference in the fraction replying “definitely not” concerned differing beliefs (Republican versus Democrat) rather than any of the standard sociodemographic differences (22).

### Policies to Reach and Sustain a High Level of Vaccination.

Our results have implications both for the specific challenge of ending the COVID-19 pandemic and for the more general problem of policy design in novel public health emergencies with a high degree of uncertainty.

What can we infer from our evidence about policies to address the pandemic? There are four main findings of our survey of German residents.

First, while there was a substantial degree of transience in opposition to both voluntary and mandated vaccinations, this is particularly true in the voluntary case. Easily accessed vaccines (39) and convincing messaging about vaccine effectiveness and the trustworthiness of public institutions (40) could persuade a significant number of the opposed to be vaccinated, consistent with our evidence on the role of changing beliefs in distinguishing between the hard-core and transient opponents.

Thus, for policy makers considering the imposition of universal mandates, a critical question is whether the transience of vaccination opposition has persisted as Germany moved, in the second half of 2021, to a hybrid of effectively mandated and voluntary policies.

The flow of new information that may account for changing vaccination attitudes over the three waves of our survey has continued since May 2021. This includes sharply increased case rates (see the *SI Appendix*, timeline and Fig. S2) to a level in early March 2022 about 10 times the maximum previously registered over the course of the survey; the diffusion of the highly transmissible Delta variant at the end of May 2021 and the associated increase in the level of vaccinations thought to be sufficient to contain the pandemic (41, 42), followed by the appearance of the immune escape Omicron variant in November 2021 resulting in reduced effectiveness of the available vaccines; the sharp increase in numbers vaccinated since May 2021 (9) and the positive effects on subsequent vaccination rates this may have had due to conformism (12, 43); the German government’s reversal of its “no mandates” commitment (6, 44), likely affecting German’s trust in public institutions; and conflicting news about vaccine effectiveness (45). There has also been a steady flow of unsubstantiated but possibly mind-changing claims on social media.

However, our data suggest that it is likely that vaccination opposition has hardened in response to the increased use of de facto mandates late in 2021 and the announced prospect of general mandates in 2022 (Fig. 1*B*, and compare Fig. 2*A* and Fig. 2*B*).

Second, our data allow a hypothetical assessment of the likely trajectory of vaccinations in Germany had they remained voluntary, with the health authorities relying on persuasion rather than enforcement. To see this, consider two thought experiments to explore the dynamics of agreement to voluntary vaccines, assuming no change in the transition probabilities estimated from our panel, that is, no change in the extent to which people adopt new beliefs and vaccination attitudes.

The small (3.3%) fraction of consistently opposed would mean that, if vaccinations were conveniently available, then, eventually, the current target rates of more than 90% (42) would be met without mandates, as happened in some areas of Germany [97% of adults in the state of Bremen were vaccinated before partial mandates were introduced late in 2021 (46)]. This would occur because most of those opposed in any single survey wave would experience a change in beliefs and vaccination attitudes toward voluntary vaccination in some subsequent period.

However, the urgent need for a substantial increase in vaccination and the fact that the German government has now adopted a policy of (at least selective) mandated vaccinations limits the relevance of this reassuring calculation. The fraction in our panel that were consistently opposed to mandated vaccinations—16.5%—suggests that a substantial number may have to be vaccinated against their will in order to reach the target vaccination levels thought to be sufficient to contain the pandemic.

Third, beliefs matter and they change. Consistent opposition and switching from opposition to willingness is not predicted by who one is (sociodemographically) but instead by one’s beliefs and attitudes, suggesting the importance of sustaining high levels of trust in public institutions and in the effectiveness of the vaccines. An example of such an attempt is the reason given for the decision by the government of Israel to allow booster vaccinations for the entire population: A prime motive was to counter the effects of widely publicized breakthrough infections among those with two doses. Prime Minster Naftali Bennett explained that “we decided the third jabs were necessary to safeguard the public’s confidence in the vaccines” (47). Social learning mechanisms such as conformism as well as conditional cooperation may also be mobilized by public policy, given that, by the end of 2021, in Germany, France, Brazil, China, the United States, and other countries, the vast majority have been vaccinated at least twice (48–54).

The fact that changes in beliefs are associated with changes in vaccination attitudes suggests a causal relationship. However, while opposition to vaccination is plausibly a consequence of distrust in public institutions and the vaccine, the reverse is also possible. Distrust in public institutions and a belief that vaccines are ineffective may be adopted unwittingly among those opposing vaccination in order to reduce discord between one’s beliefs and actions. If this reverse causal relationship were the primary one, designing policies to change beliefs would be more difficult. But, if possible, belief change could nonetheless contribute to vaccination willingness, because the cognitive dissonance entailed by coming to believe in vaccine effectiveness while remaining unvaccinated could be a strong motive to change one’s attitude about vaccinations (55).

Fourth, enforcement and persuasion are complementary policies, not alternatives. Shifting to mandates does not reduce the importance of changing beliefs. Concerning the consequences of the shift to selective and perhaps universal mandates [as, for example, in Austria and Germany (8, 44)], we find that measures that erode an individual’s “freedom” or trust in government (which could result from a shift to mandates, given previous promises of the opposite) might hinder the implementation of the entire range of anti–COVID-19 policies (11), including mandated vaccinations.

Mandates do not reduce the importance of these beliefs supporting agreement with being vaccinated. Differences in beliefs are as important under an enforced regime as in the voluntary case in explaining movement out of opposition, and much more important in explaining consistent opposition when enforced (Fig. 3 and *SI Appendix*, Table S5). Given the limits both on a government’s capacity to implement enforced vaccines and on the resources it can devote to this task, changing these beliefs—persuasion—thus remains crucial even under a vaccine mandate.

## Discussion

Support of a government’s policy appears to be based on trust and a sense of reciprocity that can be earned or squandered by the conduct of public institutions (56–60). Policy makers are right to worry that imposing mandates—especially by governments that had committed not to do so—might undermine trust in government among the targets of these mandates and beyond. But sustaining trust in government among the vast majority of adults who are already vaccinated in many countries may require more aggressive steps against the virus, including mandates. Our previous evidence (11) shows that citizens’ trust in government is associated with reduced opposition to enforcement and with greater willingness to follow the entire range of anti–COVID-19 policies such as mask wearing, social distancing, or travel restrictions.

Three final observations concern implications for the more general problem of policy design in novel public health emergencies and other societal challenges.

First, reactions to COVID-19 policies suggest some shortcomings of the economic model of policy implementation that is based on citizens as “good choosers.” In this approach, people with given (self-interested) preferences and beliefs make consistent decisions in light of the available information and, as a result, respond in the intended ways to the incentives and constraints offered by the policy maker.

The standard policy implementation model may be enriched by a more psychologically and sociologically informed perspective. This would capture the fact that people often act on the basis of moral and social concerns, for example, following herd behavior due to conformism; they may not be adept at anticipating the effect of their actions on their own well-being, leading to seemingly inconsistent behavior; and they may be slow to alter their course of action when new information is available, due to what is termed “status quo bias” (61–64). In this enriched approach, incentives and constraints remain an important policy tool, but they may be counterproductive when they alter citizens’ beliefs and preferences in adverse ways.

Second, while policy makers must be concerned that mandates and other restrictions may crowd out intrinsic or social motivations to adhere to recommended individual behaviors in the public interest, they may also aspire to design and frame policies such that constraints will crowd in these social motivations. Since Jeremy Bentham in the late 18th century, this expressive function of law has been an important element in philosophical thinking on the design of laws and constraints (65–67).

We know from empirical evidence (including experience during the COVID-19 pandemic) that this is possible (53, 68–75). For example, a player’s contributions in a public goods game were enhanced if they knew that others who free ride on their groupmates’ contributions would be punished, even if they themselves were exempt from the punishment (76). Moreover, evidence from both economic and neurological experiments suggests that adverse effects of control can be mitigated if those subject to a constraint understand its purpose (13, 17), for example, preventing free riding by others.

Third, lessons from the COVID-19 pandemic may apply more generally to other societal challenges, especially where it is difficult to enforce changes in personal behavior and social interactions that are critical to the success of public policy (for example, addressing climate change, tolerance of ethnic diversity, and racial or gender inequality). Our data and the related literature suggest that, where mandates are to be used, resistance would be lower if those affected 1) are convinced of the severity of the problem to which the mandate is addressed and 2) are convinced of the effectiveness of the mandated behavior in addressing the problem, and, moreover, 3) could see the mandate as a precondition for advancing valued social norms. In the case of COVID-19 vaccination mandates, for example, the latter includes sustaining a greater long-term level of individual freedom (instead of perceiving a mandate as a restriction). Each of these desiderata entail a high level of citizen confidence and trust in scientific, political, and public communication elites.

## Materials and Methods

### The Questions.

To study the possibility that enforcement may crowd out intrinsic or other positive motivations, it is essential not to confound social motives for an individual complying with a measure on the one hand with obedience to the law on the other. Therefore, our questions ask about the respondent’s attitude toward vaccination (“agree” in the sense of “being okay with”) and not whether a person would comply with a legally imposed and enforced vaccination policy. There are two reasons why the question on mandates was formulated this way.

First, it is likely that people might disagree with being vaccinated but still comply with it because they are legally required. This distinction is crucial to detect crowding out of intrinsic motivation due to enforcement in comparison with people’s agreement under voluntary policies. The answers to our questions in case of enforcement allow us to identify the share of people who are comfortable (“agree”) with a measure under enforcement, as opposed to the share of citizens who disagree with it and who will therefore either not adhere to a policy even though it is mandatory or follow it under enforcement but be left with heightened negative emotions like anger, aggression, frustration, and hostility toward their government.

Second, to investigate whether enforcement can succeed in implementing a measure (not the question we are asking), an appropriate survey question would inquire about behavior. However, a positive answer to this question is uninformative about subjects’ attitudes behind compliance. Instead, it measures the extent to which people state that they will obey the law. Presumably, most Germans would state in a survey that they would comply with a mandatory policy. To understand the crowding-out phenomenon, we need to elicit people’s attitudes behind their compliance behavior and not their willingness to comply with enforcement per se, which is a different research topic.

Moreover, our survey question on agreement with being vaccinated in case of voluntary implementation has a strong normative content (“strongly recommended by the government”). This serves to stress that, even in the absence of enforcement, compliance is clearly desirable. Asking the question on agreement in case of voluntary policies without stressing its normative importance would give the impression that noncompliance is equally permitted and acceptable, which is not the way in which voluntary compliance has been promoted in actual policy making.

### The Design.

To identify differential individual responses, all subjects were asked to state their agreement with being vaccinated in both cases: if it remains voluntary and if it is enforced. In a separate survey, we investigated the possibility of a demand effect due to asking a subject to answer both questions. We implemented a between-subjects design confronting respondents with only one alternative (either voluntary or enforced) and obtained results very similar to those resulting from asking each subject to answer both questions, as shown in *SI Appendix*, Fig. S12. Moreover, altering the order of the alternatives in a within-subjects design did not affect average agreement with enforced or voluntary vaccination either.

To limit a potential spillover effect—a subject answering questions in a way to minimize cognitive inconsistency—the module containing the questions on agreement with being vaccinated and the module containing the questions about vaccine effectiveness were separated by a module unrelated to vaccination.

### The Panel.

The questions were embedded in an ad hoc online survey on COVID-19 initiated by the Cluster of Excellence “The Politics of Inequality” at the University of Konstanz.

Their predefined target sample size was 4,700 subjects in the first wave. All first-wave participants were invited to the second and third waves. To reach roughly 4,000 subjects in each cross-section survey, in addition, new participants were invited based on the representative sampling criteria. Participants were recruited from a commercial online access panel administered and remunerated by the survey provider respondi, which usually conducts market research. Membership of the respondi survey pool and participation in its surveys is voluntary and follows a double opt-in registration process. Participation is incentivized with tokens that can be exchanged for goods. Given this material incentive, people registered there are unlikely to have atypical intrinsic or social motivation relevant to the subject matter of the survey. This is important because, otherwise, voluntary participation in the survey might create a sample bias in favor of voluntary policies.

The panel was implemented and run by the surveyLab at the University of Konstanz. The first wave was conducted from April 29 to May 8, 2020, the second wave was from October 28 to November 6, 2020, and the third wave was from May 5 to May 18, 2021. As follow-up waves were not planned initially, the survey was never announced to respondents as a longitudinal study. Our questions on agreement with being vaccinated in case it is voluntary or enforced were part of several modules on topics related to COVID-19. Invited participants self-selected into the online panel titled “Living in exceptional circumstances,” and subjects were not aware of the specific topic of any module (including ours) when agreeing to participate.

Before and after the modules, respondents answered a series of questions on sociodemographics and other controls. Basic demographics answers were mandatory, in particular, for the questions concerning the sampling criteria. All other questions were voluntary, and subjects were free to quit the survey at any time. Median response time in the first, second, and third waves of the survey was 14, 18, and 18 min.

### Participants.

Participants were required to be 18 y of age or older, German speaking, and residents of Germany. The quota reflected the resident population in terms of (the marginal distributions of) age group, gender, education, and region. As East and West Germans have been shown to differ in their motivations in case of voluntariness versus enforcement (11, 33) and as there are many fewer East Germans than West Germans, double quota for East Germany were used. All results reported in the paper are based on unweighted observations. Using sample weights to achieve full representativeness has little effect on our main findings (*SI Appendix*, Table S3). The mean age of the panel sample was 53 y (SD: 14 y), and 47% were female (all demographic variables are reported in *SI Appendix*, Table S2).

The following exclusion criteria were defined by the surveyLab: dropout during the survey, nonsense responses to open questions, speeders, and straight lining. Exclusions were performed by the surveyLab based on an independent standard quality check, without any involvement of the authors of this article. Moreover, we use list-wise exclusion of subjects with missing data in the variables used for the regressions. See *SI Appendix*, Table S1 for details.

### Ethics Approval.

This study was approved by the Ethics Committee of the University of Konstanz, IRB 20KN09-006. All subjects provided informed consent. We have complied with all relevant ethical regulations.

## Supplementary Material

Supplementary File

## Data Availability

The anonymized survey data and code files to replicate the results of the paper have been deposited at GESIS SowiDataNet datorium (German Data Archive for the Social Sciences) and are available at https://doi.org/10.7802/2375 (77).
