# Supplementary material for: Opposition to voluntary and mandated COVID-19 vaccination as a dynamic process: Evidence and policy implications of changing beliefs
Source: Proc Natl Acad Sci U S A. 2022 Mar 22;119(13):e2118721119. doi: 10.1073/pnas.2118721119 (PMC9060490; doi:10.1073/pnas.2118721119)
Supplement: Supplementary File [file pnas.2118721119.sapp.pdf]

PNAS
www.pnas.org

Supplementary Information for

Opposition to voluntary and mandated COVID-19 vaccination as a dynamic process: Evidence and policy implications of changing beliefs

Katrin Schmelz* and Samuel Bowles

*Corresponding author

Email: katrin.schmelz@uni-konstanz.de

This PDF file includes:

Supplementary Text
- Main survey question on agreement with being vaccinated (original screenshot).
- Timeline of important news on COVID-19 in Germany before and during the three waves of the survey.
- Evidence that the observed small share of those consistently opposed to vaccination is not due to response error (incl. Fig. S1).

Figures S2 to S12
- Fig. S2. Timing of the panel relative to the course of the COVID-19 pandemic in Germany.
- Fig. S3. Percentage of total population vaccinated at least once in Germany.
- Fig. S4. Cumulative distribution of agreement with being vaccinated if voluntary or enforced, among adults in Germany, May 2021
- Fig. S5. Consistency and change in the responses of those undecided.
- Fig. S6. Comparison of the German panel and the representative wave 3 cross section and the German and US responses to the Kaiser Family Foundation questions.
- Fig. S7. The distribution of consistently opposed, consistently willing, consistently undecided and inconsistent responses across the 3 waves among older East and West Germans.
- Fig. S8. The fraction of difference between the consistently opposed and others explained by sociodemographic differences alone and the full model including beliefs (Tjur's R^2).
- Fig. S9. Fractions of older East and West Germans who were opposed to and agreed with being vaccinated in at least one wave of the panel.
- Fig. S10. Logit and linear regressions predicting consistent opposition to vaccination, that is responding 0 or 1 in all three survey waves.
- Fig. S11. Logit and linear regressions predicting those who switched from opposing vaccinations in wave 2 (levels 0 or 1) to supporting vaccinations in wave 3 (levels 3 or 4).
- Fig. S12. Agreement with being vaccinated if voluntary and if enforced in within-subjects and between-subjects designs.

Tables S1 to S7 (see the next page)

Table S1. Number of participants in the three cross-section waves of the survey and the panel, dropouts, and exclusion criteria.
Table S2. Socio-demographics for the representative cross-sections and the panel waves.
Table S3. Comparing opposition to vaccination with and without sample weights in the panel sample.
Table S4. Explaining the variables in the regressions.
Table S5. Goodness of fit predicting consistent opposition to vaccination and movement from opposition to agreement between waves 2 and 3.
Table S6. Transition matrices and stationary distributions for the full 5-point Likert scores.
Table S7. Transition matrices and stationary distributions for a 3-state process (opposed, undecided, and willing).

References

Main survey question on agreement with being vaccinated (original screenshot)

Inwieweit sind Sie einverstanden, sich selbst impfen zu lassen, wenn ...

	Überhaupt nicht einver- standen 0	1	2	3	Voll und ganz einverstanden 4
... die Impfung von der Regierung dringend empfohlen wird, aber freiwillig bleibt?	○	○	○	○	○
... die Impfung von der Regierung verpflich- tend eingeführt und kontrolliert wird?	○	○	○	○	○

Timeline of important news on COVID-19 in Germany before, during and after the three waves of the survey

The timeline below shows that while the state of the pandemic differed substantially between the periods of the three surveys – cases were low and falling in May 2020, and high and rising in November 2020, and high and falling in May 2021 – the information available to the public about the vaccines and the vaccination policies likely to be adopted by the German government was not substantially different.

All information reported below is taken from major German newspapers on the indicated dates.

News with respect to COVID-19 vaccines are highlighted in blue.

January 2020

27 January. First German infected with COVID-19.

February 2020

24 February. COVID-19 has arrived in Europe as an epidemic - serious situation in Italy.

March 2020

2 March.

- Recommendations: sneeze into the elbow, wash your hands regularly, avoid handshakes.

10 March. Recommendation: cancellation of all major events with more than 1,000 participants.

16 March. Press conference by Chancellor Merkel on anti-COVID-19 lockdown measures:

- Schools and day-care centers are already closed in most of the federal states, others will follow.
- Shops are to close – except for supermarkets, pharmacies, drugstores, petrol stations and hairdressers.
- Restaurants may open only between 6am and 6pm.
- Places of worship, playgrounds, sports facilities, bars, clubs, theatres, cinemas, concert halls and museums will be completely closed.
- Restrictions on travel, borders are closed.

18 March.

- Historic TV address of Chancellor Merkel. In an urgent appeal, she calls on the population to act in solidarity and responsibility. “Social contacts must be minimized.” “This is serious. Take it seriously, too.”
- EU imposes entry ban.
- Robert-Koch-Institute (RKI): coronavirus vaccine realistic by spring 2021.

20 March. Bavaria imposes state-wide curfew.

22 March.

- Federal and state governments agree on strict restrictions on exit and contact. Citizens may only be in public areas with a maximum of one person who does not live in the same household and must keep at least 1.5m distance.
- Restaurants and pubs may only offer take-away food. Hairdressers must close.

28 March. Infection Protection Act comes into force (i.e., the government is entitled to restrict fundamental rights).

April 2020

1 April.

- The nationwide contact restrictions are extended until 19 April. People should generally refrain from private travel and visits - including those by relatives.

15 April.

- The severe restrictions on contact will be extended until 3 May.
- Stepwise reopening of schools on May 4.
- Restaurants, bars and pubs are to remain closed as before.
- Major events will also remain prohibited until at least 31 August.

16 April. Government recommends to wear community masks when shopping and in public transport.

17 April. Germany survived the first wave of Covid-19 well, gradually returning to normality.

20 April. First cautious relaxations of the anti-Covid-19 measures come into force.

22 April. Paul-Ehrlich-Institute has approved the first clinical trial of a corona vaccine to be tested in Germany.

27 April. Wearing face masks is mandatory in all federal states for shopping and public transport.

SURVEY WAVE 1 STARTS ON 29 APRIL, 2020

29 April.

- First test subjects of German vaccination study have been injected.
- Minister of Health Spahn: enforced vaccination will not be necessary, voluntary willingness to get vaccinated is sufficient.

30 April. Chancellor Merkel is consulting with the heads of the federal states on how to proceed:

- Contact restrictions remain in force for the time being. Citizens are to keep a minimum distance of 1.5 meters in public and only stay there alone, with another person not living in the household or with members of their own household.
- Playgrounds are to be permitted again under certain conditions.
- Community worship services should be allowed again with rules on distance and hygiene.
- Schools and daycare centers: no changes, federal and state governments want to discuss this in more detail on 6 May.
- Restaurants, hotels and cafés will remain closed.
- No changes for the time being with respect to store openings.

May 2020

2 May.

- Some federal states relax some measures, contrary to the federal and state agreements from 30 April.
- Demonstrations against the corona restrictions.

3 May. Minister of Health Spahn thinks that Germany will stick to voluntary vaccinations. Some other prominent political figures, e.g. Bavaria's Prime Minister Söder, express that they would favor enforcement.

4 May. Hairdressers reopen.

5 May. RKI head Lothar Wieler pleads for flexible relaxations of the measures in Germany, depending on the local incidence of infections.

6 May. The federal and state governments have agreed on further relaxations, though contact restrictions remain largely in place:

- Families from two different households can meet.
- Schools: gradual reopening.
- Daycare: remain closed.
- Hotels and restaurants: gradual reopening, subject to hygiene and distancing conditions.
- Shops: all shops can open but have to meet hygiene and distancing requirements. Wearing masks is mandatory.
- Districts with more than 50 infections per 100,000 inhabitants within the last 7 days will have to return to stricter restrictions immediately.

8 May.

- Three districts already break the negotiated upper limit for new infections.
- Growing resistance against the corona measures. Critics say that the restrictions of basic rights are too severe.
- Many fear a compulsory COVID-19 vaccination - for a vaccine that does not yet exist.

SURVEY WAVE 1 ENDS ON 8 MAY, 2020

16 May. RKI and government confirm that relying on citizens' sense of responsibility, vaccinations will be voluntary.

August 2021

11 August. Putin announces Russia's approval of the world's first COVID-19 vaccine *Sputnik* to the media.

September 2020

15 September. Health Minister Spahn has reiterated that COVID-19 vaccination will not be compulsory.

17 September. Many Germans hesitant – will voluntary vaccination work?

24 September. According to the German Standing Committee on Vaccination (STIKO), it is unclear whether enforcement of COVID-19 vaccinations will be beneficial. Someone who absolutely does not want to be vaccinated will always find ways to get around it.

October 2020

1 October.

- *CureVac* expects vaccine in the first half of 2021. Second phase of clinical trial started this week.
- Minister of Health Spahn: Prioritized vaccination may be needed, such that, for example, health care workers and at-risk groups get the vaccine first.

2 October. Research is being conducted on many possible COVID-19 vaccines worldwide. A German company also has a promising candidate in the decisive test phase. When will the vaccination come?

5 October. Experts at the RKI are dampening hopes for a quick return to normality after a vaccine is admitted. This is because it will initially only be available in limited quantities.

6 October.

- *AstraZeneca* announces that it will provide vaccine data from trial series by the end of the year.
- European Medicines Agency (EMA) approval process for *BioNTech/Pfizer* vaccine starts.

7 October. The U.S. Food and Drug Administration has tightened the requirements for emergency approval of a COVID-19 vaccine - apparently against the will of the White House.

8 October.

- The number of infections in Germany is increasing dramatically and almost reaches the mark of 4,000 new cases in one day. Alarming thresholds are exceeded in Berlin and Frankfurt.
- Research Minister Anja Karliczek expects widespread COVID-19 vaccination to be possible from mid-2021. Currently, three companies are receiving federal funding for vaccine development.

10 October. Although Chinese vaccine candidates are still in final testing phase, hundreds of thousands of Chinese are already being vaccinated. Still unclear how safe and effective Chinese vaccines actually are.

12 October.

- One district exceeds the critical mark of 50 new infections per 100,000 inhabitants within seven days.
- Federal Health Minister Spahn expects that vaccinations in Germany can begin in the first quarter of 2021. People with pre-existing health conditions, the elderly and health and care workers will be targeted first.

14 October. The federal and state governments agree on new containment policies in hotspots:

- In regions with 50 or more new infections per 100,000 inhabitants within seven days, private parties are to be limited to a maximum of ten people and two households. There is to be an 11 p.m. curfew for restaurants.
- In regions with 35 new infections per 100,000 inhabitants within seven days, the mask requirement is also to apply where people gather more closely or for longer periods.

15 October.

- The number of new infections in Germany rises to over 6,000 within a day.
- Reactions to the federal-state resolutions on infection control are polarized.
- Bavaria issues its own, stricter regulations.
- Courts in two federal states overturn a controversial ban on accommodation for guests from high-risk regions within Germany, while other state governments suspend it.

17 October.

- In view of the sharp rise in new infections, Chancellor Merkel is calling on the population to reduce contacts as far as possible.
- *BioNTech/Pfizer* has already started mass production of a vaccine.
- *Pfizer* will apply for emergency approval in the U.S. by the end of November.

13-18 October. Reports of fake news claiming that vaccination would be compulsory and fact checks that this is not true.

19 October. The first district in Germany has again very strict exit and contact restrictions.

20 October. *Moderna* says its vaccine could receive U.S. emergency approval in December.

23 October.

- German Federal Ministry of Health reaffirms expected COVID-19 vaccination start in early 2021. Previously, there have been media reports about a possible vaccination start this year.
- Health Minister Spahn: there will be no compulsory vaccination.

25 October. COVID-19 opponents are mobilizing not only on social media, but also with flyers containing misleading and false claims.

26 October. *AstraZeneca*'s vaccine appears to elicit a "robust immune response" among the elderly. Results will be published soon.

SURVEY WAVE 2 STARTS ON 28 OCTOBER, 2020

28 October. Second lockdown "light" announced in Germany, lasting for the entire month of November:

- Restaurants and houses of culture close, no tourism.
- Contact restrictions of at most 10 people or two households.
- Schools and daycare remain open.
- Commerce and business will keep running.

29 October.

- German Standing Committee on Vaccination (STIKO) announces that Germany will not be fully vaccinated (which meant vaccinated twice at that time) until 2022.
- According to an article in The Lancet by the chair of the U.K. Vaccine Task Force, upcoming vaccines "probably won't be perfect" and "may not work for everyone".

30 October. EU spreads optimism, is the vaccine coming soon? European Parliament is discussing December/January as start dates for vaccinations. Health professionals are to be vaccinated first. But the first vaccines may not fully protect everyone.

31 October. Paul Ehrlich Institute expects first vaccine approvals in early 2021. However, the approval of a vaccine does not mean that it will be immediately available to the entire population.

November 2020

1 November.

- A suggestion for the national vaccination strategy will be presented in the coming week. It will address an ethically sensitive question: who comes first, who comes last?
- Nearly one in ten German health departments complains of being overwhelmed.

2 November. Lockdown "light" starts.

3 November.

- The vaccine of the Germany-based company *CureVac* has successfully passed the first clinical test phase. The 250 subjects showed a responsive immune response and good tolerability.

5 November. Despite tight contact restrictions, COVID-19 infections in Europe continue to rise rapidly.

6 November.

- German government classifies almost all of Europe as a risk area. Hospitals in Germany are preparing contingency plans for the treatment of Covid-19 patients.
- Mutation of SARS-CoV-2 found on a mink farm in Denmark, concern that the vaccines developed so far might not work against mutations.

SURVEY WAVE 2 ENDS ON 6 NOVEMBER, 2020

9 November.

- *BioNTech/Pfizer* announce that a vaccine will be available soon, 90% success rate.
- Chancellor Merkel announces that the lockdown policies will become more stringent instead of relaxed.
- Recommendations from scientists: "A general vaccination mandate should be ruled out"

16 November. *Moderna* announces a new vaccine with 94% effectiveness.

December 2020

14 December. First vaccinations in the U.S.

18 December. Woman in the U.S. suffers allergic shock after Corona vaccination.

21 December.

- BioNTech/Pfizer's Comirnaty vaccine received conditional marketing authorization in the EU.
- Side effects of vaccination: unpleasant, but not dangerous.

24 December. First case of the more infectious Alpha variant in Germany (which became the dominant variant in Germany until May 2021).

26 December. First vaccinations take place in Germany (Biontech/Pfizer).

January 2021

5 January. Lockdown extended until end of January.

10 January. Success after three weeks: 20 percent of all Israelis already vaccinated.

12 January. Bavarian premier Söder calls for the German Ethics Council to examine mandatory vaccination for certain professions like health care staff.

13 January. Several politicians, including Health Minister Spahn, criticize Söder's push. Federal Minister of Justice Lambrecht again speaks out against mandatory vaccination. "The federal government has clearly stated that there will be no compulsory vaccination against COVID-19. The word of the federal government is something that you can count on. […] If people are convinced of the safety and effectiveness of the vaccination, the vast majority will get vaccinated."

19 January.

- The federal and state governments have agreed on a longer lockdown and a stricter mask requirement. Chancellor Merkel justifies this with the virus mutation.
- Are the rapidly developed vaccines really safe? What normal vaccination reactions should I expect? What are the side effects?

21 January. Deaths after Corona vaccinations (Biontech/Pfizer): Norway urges caution.

27 January. U.S. continues to outpace Germany in vaccination progress.

29 January. Vector vaccine from AstraZeneca/Oxford approved in Germany.

February 2021

3 February. Study by Oxford scientists: AstraZeneca vaccine significantly reduces virus transmissibility.

11 February. The federal and state governments have not been able to agree on a detailed stage-by-stage plan on openings, but at least they have agreed on a first concrete threshold for a gradual ramp-up of the economy and for openings.

18 February. AstraZeneca vaccine: "We didn't expect 40 percent side effects".

22 February. Swedish regions stop vaccination with Astrazeneca - "Surprising" accumulation of side effects.

March 2021

4 March. The lockdown will remain in place until March 28, but with some relaxations. Depending on the incidence, there will be further relaxations, or an emergency brake.

11 March. Vector vaccine from Johnson & Johnson approved in Germany.

15 March. Germany suspends vaccination with *AstraZeneca*. All newspapers report about worrying side effects of the *AstraZeneca* vaccine.

25 March. *AstraZeneca* vaccinations can continue following a new assessment by the European Medicines Agency.

April 2021

1 April. STIKO advises younger people not to take AstraZeneca as second dose.

13 April. Thrombosis cases: USA to suspend vaccination with Johnson & Johnson for the time being.

14 April. "Serious" side effects: Denmark stops Astrazeneca vaccinations for good.

16 April. Head of Biontech/Pfizer assumes third and annual vaccine doses.

21 April.

- Brain thrombosis after *AstraZeneca* vaccination.
- The German parliament has passed the nationwide COVID-19 "emergency brake". The federal government can thus significantly expand its powers.

29 April. Israel investigates cases of heart muscle inflammation after vaccination.

May 2021

4 May. The Federal Cabinet has approved the ordinance on the rights of vaccinated and recovered persons. Among other things, there are plans to relax the contact restrictions and curfews.

SURVEY WAVE 3 STARTS ON 5 MAY, 2021

5 May. Hopeful words from the Chancellor's Office: Relaxations of lockdown policies in May and Vaccine for all in July.

6 May. Israel: Biontech's vaccine also protects against asymptomatic infections and the British variant.

7 May.

- According to Federal Health Minister Spahn, the third wave of the pandemic seems to be broken. However, the case rates in Germany remain at a high level and must decrease. Prioritization of AstraZeneca's vaccine will now be completely abandoned.
- Horrible pictures from India given the new variant.

9 May. New regulations in force: relief for vaccinated and convalescent patients.

10 May.

- One third of Germans vaccinated at least once.
- *AstraZeneca*: Earlier second vaccination lowers COVID-19 immune protection.
- WHO: Indian (Delta) variant "worrying".

11 May. Thousands attempt to jump the queue to get vaccinated.

17 May. Health Minister Spahn plans to lift prioritization: vaccination possible for all as of June 7.

SURVEY WAVE 3 ENDS ON 18 MAY, 2021

26 May. Vaccination recommendation applying to all children unlikely.

27 May.

- Researchers think they have found the reason for side effects (thrombosis) of vector vaccines like Astra Zeneca. If they are right, producers could fix the problem.
- More second than first vaccinations. Vaccination rate rises to 41.2 percent.

31 May.

- Anti-COVID-19 emergency break could expire at the end of June. CDU/CSU and SPD are considering not extending the central policies to contain the pandemic. In the event of another wave, the federal states would then be responsible again.
- First vaccine for children approved in the EU (Biontech/Pfizer).

June 2021

1 June. New study suggests that the positive effects of lockdown and emergency brake are significantly overestimated.

10 June. STIKO recommends vaccination for 12 to 17 years old children with pre-existing health issues.

17 June. CureVac vaccine not as effective as hoped for (efficacy of 47 percent).

26 June. The Delta variant is now most common in Germany. Vaccines protect less against this variant.

July 2021

2 July. Following STIKO recommendation: Spahn promotes cross-vaccination.

12 July. Member of the Ethics council calls for mandatory vaccination of teachers and educators.

23 July. Rising case rates. Virologist Drosten warns of "winter wave".

25 July. Debate about pressure on vaccination opponents, Minister President Armin Laschet against discriminating the unvaccinated.

August 2021

11 August.

- Uncertainty about actual vaccination rate, is it significantly higher? RKI puzzled by "uncertainty" in reporting.
- There should be no compulsory vaccination in Germany, but there is discussion about whether vaccinated people should have more freedom than others. What other means could be used to increase the vaccination rate? And would compulsory vaccination even be constitutionally enforceable?

16 August. STIKO recommends vaccination for everyone from 12 years on.

20 August. Spahn considers booster vaccinations for all.

September 2021

2 September. Drosten anticipates society-wide contact restrictions. "Can't go into fall with this vaccination rate".

8 September. We are in a pandemic of the unvaccinated. Most COVID-19 patients in the intensive care units are not vaccinated.

10 September. STIKO recommends vaccination for pregnant women.

13-19 September. Germany-wide vaccination week to increase vaccine uptake.

19 September. The federal state of exemption should end, according to Health Minister Spahn. While some are now hoping for a "Freedom Day" soon, others think the move sends the wrong signal.

20 September. Members of "pathology conference" spread unsubstantiated claims about COVID-19 vaccinations and deaths.

24 September. STIKO recommends booster vaccination only for high-risk patients.

28 September. Pediatricians want mandatory vaccination for teachers and health care workers.

October 2021

10 October. Experts have noted a slight increase in heart problems after Corona vaccination in young men. Still, they emphasize: The benefits of vaccination clearly outweigh the risks, they say.

November 2021

4 November. Despite a whistleblower's allegations about a study on the Biontech/Pfizer vaccine, experts say there is no reason to doubt the vaccine's effectiveness.

6 November. Death after vaccination: twelve-year-old suffered from heart inflammation.

9 November. Virologist Drosten: We are in a real emergency situation. Measures such as free citizen testing or a broadly deployed 2G model do not promise sufficiently rapid success in breaking the fourth wave. From a scientific perspective, contact restrictions and closing vaccination gaps should also be considered.

10 November. STIKO changes vaccination recommendation: because of complications (heart muscle inflammation), No Moderna vaccine for people under 30 years and those pregnant.

11 November. Drosten: boosters are the way to go. “We don't have a pandemic of the unvaccinated.”

12 November.

- Study showing that 90 percent vaccination rate necessary.
- Patient advocates warn against mandatory vaccinations for health care workers.

17 November. Epidemic emergency: Merkel wants to link COVID-19 policies to hospital occupancy rates.

18 November.

- Merkel: “The situation is highly dramatic”.
- New federal-state-resolutions: vaccination mandate for health care workers, “3G” rule (vaccinated, recovered or tested) in public transport and at the workplace.
- STIKO recommends booster for everyone 18 years and older.

19 November. Experts criticize decisions of the Conference of Minister Presidents from 18th November. More stringent measures needed in states with low vaccination rates.

22 November. STIKO recommends that people under 30 years of age should be vaccinated only with Biontech/Pfizer and no longer with Moderna.

24 November. New variant Omicron detected in South Africa. This seems to be an immune escape variant, being more infective among those recovered than the Delta variant and potentially making the vaccines less effective. Still, getting vaccinated is the best option.

27 November. First two Omicron cases detected in Germany

December 2021

2 December.

- Nationwide restrictions (lockdown) for the unvaccinated.
- The “2G” (vaccinated or recovered) and “3G” (vaccinated, recovered or tested) rules are mandatory in many areas.
- Fake news about intensive care units.
- Most COVID-19 vaccines will work as boosters.

3 December.

- Several federal states are beginning to implement the stricter policies. In doing so, some are going beyond the resolutions of the federal government and the state premiers.
- Some Ministries of State have commissioned expert opinions on a possible general COVID-19 vaccination mandate. The report concludes that such a vaccination mandate would be possible under certain conditions.

Evidence that the observed small share of those consistently opposed to vaccination is not due to response error

Response error (random mistakes in recording one's answer to a survey item) may generate inconsistency across the waves of the panel, falsely appearing to document movement out of and into opposition to vaccination. We showed in the text that from our data we can infer that even if there were to have been no change in vaccination attitudes, so that error is the only source of inconsistency in our data, we would still find a very small share of (by assumption consistent) opponents to voluntary vaccination. Our data give us the following additional reasons to think that it is unlikely that the small fraction that we observe to be consistently opposed to vaccination could be primarily the result of response error.

1. *Those favoring vaccinations are much less inconsistent*. If lack of consistency among those opposed to voluntary vaccinations were due to response noise, then we would expect to observe similar lack of consistency among those favoring vaccinations. As is evident in Fig. 2 in the paper, this is not the case, as can also be seen from Fig. S1 below. The latter figure presents individuals' level of agreement with being vaccinationed in the next survey wave depending on their level of agreement in the previous wave. The black bars refer to the consistent respondents (those who responded the same way in two consecutive waves). For example, the top panel captures those strongly opposed to vaccination (agreement level 0) in wave 1 (left) and wave 2 (right). Only 37 percent of them are consistent in the second and 38 percent are consistent in the third wave. In contrast, among strong supporters (level 4 in the previous wave, bottom panel), 63 percent (left) and 75 percent (right) were consistent in the next wave.

Another look at the evidence: The within-individual variance of responses over the 3 waves is 2.34 for those who strongly opposed voluntary vaccinations in at least one of the waves and only 0.97 for those who strongly favored vaccinations at least once.

If one were to arbitrarily assign different response error rates to different types of attitudes towards vaccination, one could probably reconcile our survey results with the hypothesis that there is no inconsistency among those opposed and that there are a great many of them. But we doubt that there is a plausible account of the source of error in responses that would explain the difference in consistency of the opposed and those supporting vaccinations. The same applies to the further evidence below.

2. *Reported changes in vaccination attitudes are associated with plausible changes in beliefs*. We find the statistical associations shown in Fig. 3B and Fig. S11, for wave 2 and wave 3 (for which we have adequate data on changes in beliefs), to be plausible, suggesting that those changing their response from one wave to the next have likely changed their attitude towards vaccination rather than recorded their beliefs erroneously.

3. *Opposition to mandated vaccinations is much less inconsistent.* If response noise were the reason for the small number of consistently opposed (responding 0 or 1) to voluntary vaccinations then we would expect to find a similar noise-induced appearance of inconsistency in other parts of the survey, e.g. for the case in which vaccinations are required by law. But this is not what we find. The persistent 3.32 percent represent a fifth (19.64%) of the average opposition to voluntary vaccination (3.32% persistent across the three waves *divided by* 16.90%, the average level of opposition over the three waves, *equals* 19.64%). By contrast, in case of enforcement, half of the average opposition is persistent across the three waves (16.50% persistent opposition *divided by* 33.17% average opposition *equals* 49.75%). Thus, the volatility of opposition is peculiar to the voluntary case, which would be unlikely if the observed inconsistency were largely noise.

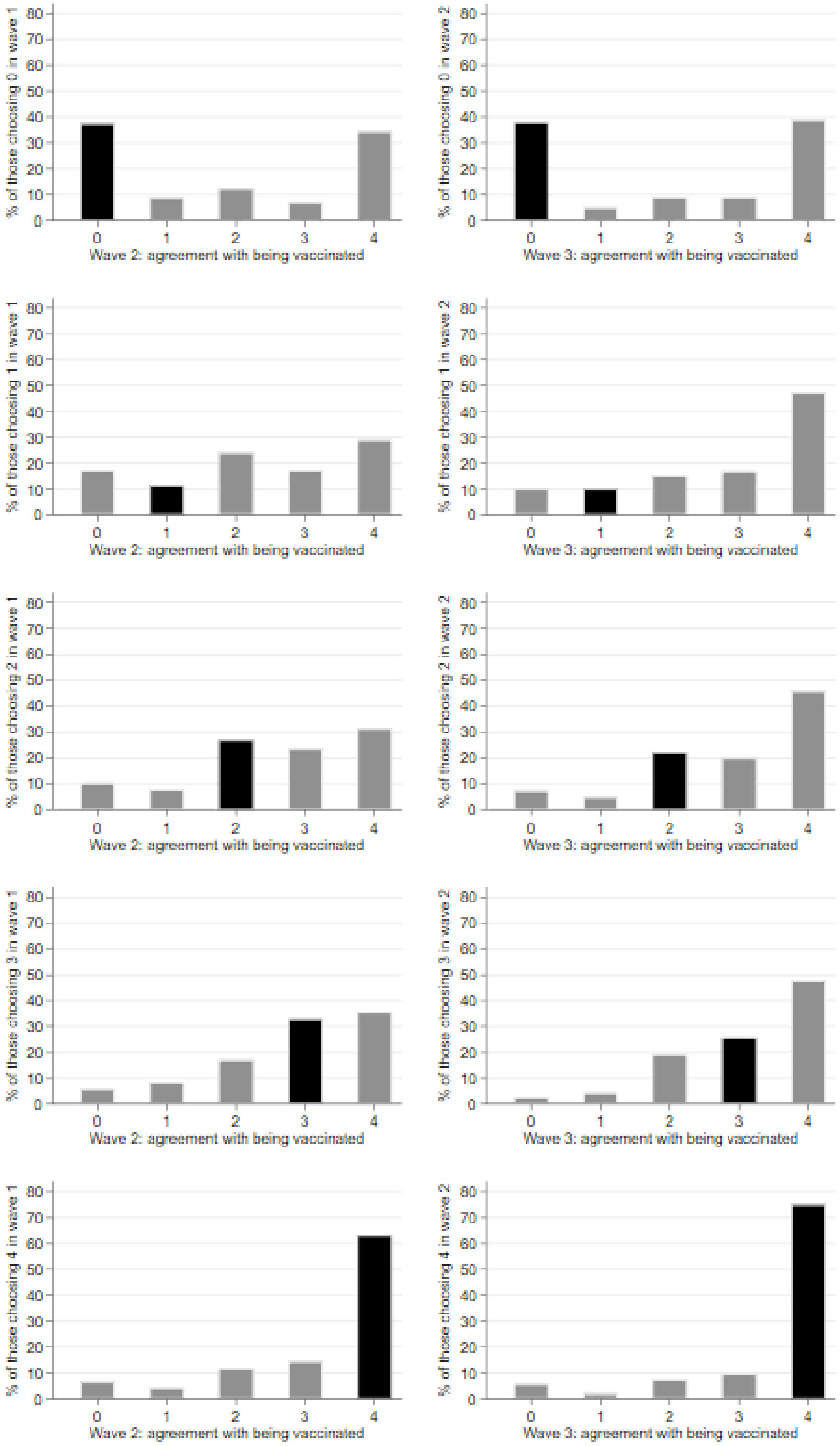

Fig. S1. Agreement with being vaccinated voluntarily given a respondent's agreement level in the previous survey wave. The left set of panels refers to the second wave and the right panel refers to the third wave. The five rows correspond to the five levels of agreement, ranging from 0 to 4. For example, the top right panel shows that more than half of those who strongly opposed vaccination in wave 2 switched to either being undecided (level 2, 9%) or supporting vaccination (levels 3 and 4, 48%). (n=2,018 for waves 1 and 2 and n=1,890 for wave 3, as those vaccinated twice in the third wave were not asked this question.)

4. *Those undecided change asymmetrically towards favoring vaccination, rather than randomly.* We can also make inferences based on those who were undecided (responding with a 2 on the Likert scale that ranges from 0 to 4) in an earlier wave and responded differently in a later wave. If inconsistency of those opposed to voluntary vaccination were mainly due to noise, we would expect their later responses to be distributed symmetrically (equal numbers changing to oppose and to favor). But as the third row of Fig. S1 shows, when the undecided change, they do so asymmetrically, overwhelmingly switching to agreement (between the first and second waves 3 times more switch to favoring than to opposing; between the second and third waves 5.4 times more switch toward favoring).

5. *Our survey predicts the subsequent level of vaccinations*. If the data were very noisy it would be unlikely that the fraction reporting willingness to be vaccinated at the beginning of the vaccination campaign (May 2021) would so accurately predict those who were actually vaccinated in the succeeding two months (vaccines became widely available only in summer) and the slowdown in vaccinations after reaching that level.

We conclude from these 5 points (and from the hypothetical example in the paper) that it is unlikely that the apparent inconsistency of those who record opposition to voluntary vaccination could be primarily the result of response noise.

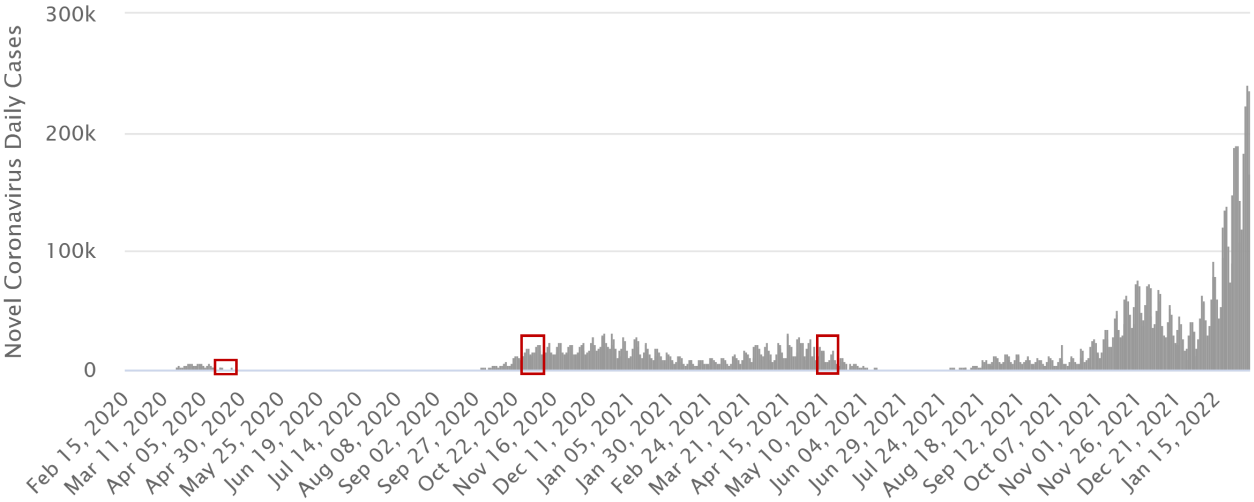

Fig. S2. Timing of the panel relative to the course of the COVID-19 pandemic in Germany. The red frames indicate the time frames of the three waves of our survey. The chart is taken from worldometers.org (1).

Vaccination rate for adult population based on the vaccination rate for the entire population in Germany and its evolution over time.

Fig. S3 shows the percentage of the total population vaccinated with at least one dose. This number grew at the average rate of 1.37% per day between the midpoint of our May 2021 survey (May 11th) wave and July 23th when the estimated fraction of adults vaccinateed reached 73%, and at the rate of 0.16% daily between then and November 18th when vaccination mandates for health care workers were announced in Germany (see the timeline).

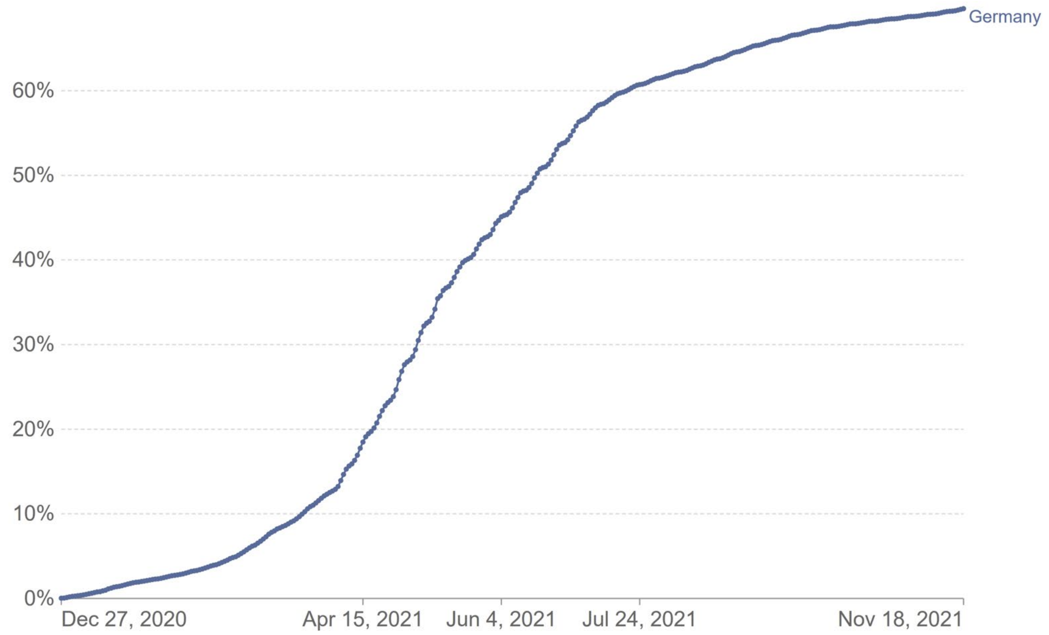

Fig. S3. Percentage of *total* population vaccinated at least once in Germany. Source: OWID (2). The percentage of adults vaccinated is substantially higher (see above). With the announcement of required vaccinations for health sector workers on 18 November 2021 Germany transited to a more mandated regime.

In Germany, 13.75 million are younger than 18 (3), which corresponds to 16.5% of the total population of 83.1 million. The 61% vaccination rate based on the total population end of July (2) then corresponds to 73% of the adult population (0.61/(1 - 0.165) = 0.73).

Fig. S4 shows the cumulative distribution of the willingness to be vaccinated (excluding those few vaccinated twice). For example, the blue and red lines show that 55% and 36%, respectively, of respondents “fully agreed” with being vaccinated if it is voluntary and enforced, respectively. Opposition (levels 0 and 1) was expressed by 15% if voluntary and 35% if enforced (that is, the final two steps in the graph).

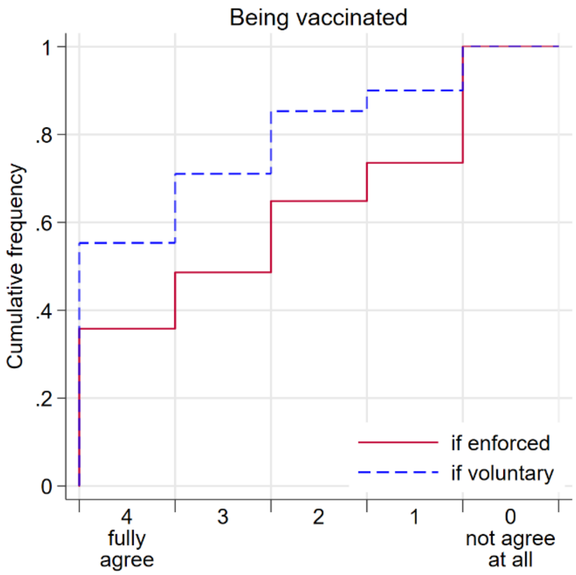

Fig. S4. Cumulative distribution of agreement with being vaccinated if voluntary or enforced, among adults in Germany, May 2021. (Data from cross-section wave 3, n=3,750.)

Fig. S5 complements Fig. 2 in the paper, showing that from one wave to the next most of the undecided became willing in the case of voluntary vaccinations (left). In the case of enforcement (right), a substantial fraction of the undecided became opposed from wave 1 to wave 2, while the majority of the undecided became willing from wave 2 to wave 3.

Most of the undecided changed their minds, 65 percent switching to willing from the second to the third wave and just 12 percent becoming unwilling if vaccinations are voluntary. In case of enforcement, the dynamics of the undecided have changed. Earlier in the pandemic, 46 percent of the undecided subsequently became opposed and only 29 percent changed their mind towards willingness from wave 1 to wave 2. But from wave 2 to wave 3, 55 percent of the previously undecided became willing and 20 percent became opposed.

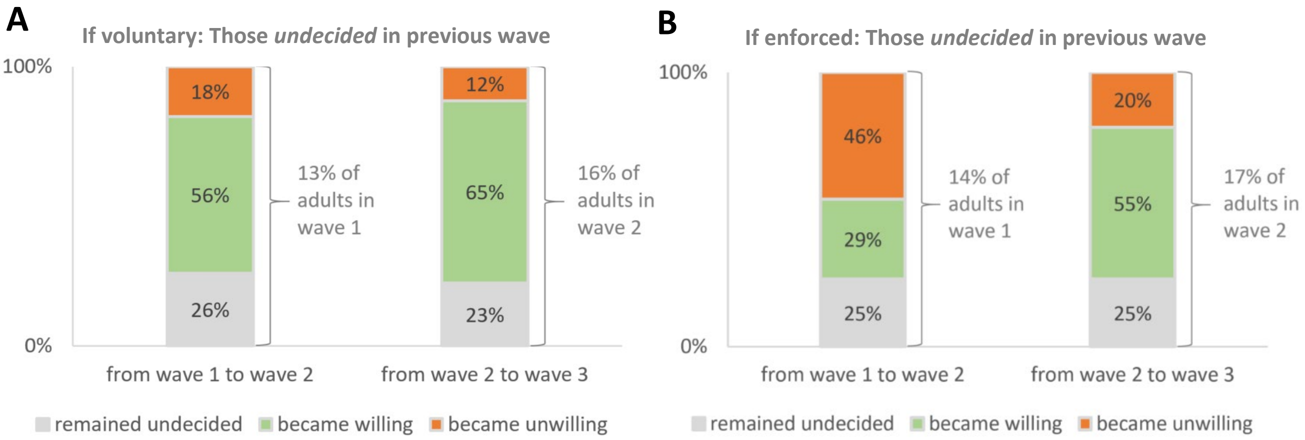

Fig. S5. Consistency and change in the responses of those undecided. How the undecided (responding with Likert scale answer 2) change from one wave to the next in the cases of voluntary and enforced vaccination.

A note on the representativeness (on vaccination attitudes) of the German panel and a comparison with U.S. cross-section data.

Fig. S6 provides information about the extent to which our German 3-wave panel is representative of the German population (comparing the first and second row) with respect to their vaccination attitudes and how different the German population sampled is from the Kaiser Family Foundation cross-section sample in the U.S. (comparing the second and third row). We cannot compare German and U.S. populations on changes in individual's vaccination preferences because no U.S. data equivalent to our survey exist.

Comparing the upper two rows in Fig. S6 shows that the distribution of responses is very similar, suggesting that our panel is not unrepresentative of the German population in this respect. The second comparison (second and third rows) shows that the German public in May 2021 differed from the U.S. in March 2021 (somewhat fewer already vaccinated, more "wait and see"), but the differences are not substantial. Using the numbers from Fig. S6, as a fraction of those unvaccinated, those who wanted to "wait and see" were 0.25 in the U.S. and 0.18 Germany, those "definitely not" getting vaccinated 0.19 and 0.17, respectively, and those who would get vaccinated "only if required" 0.10 and 0.08, respectively.

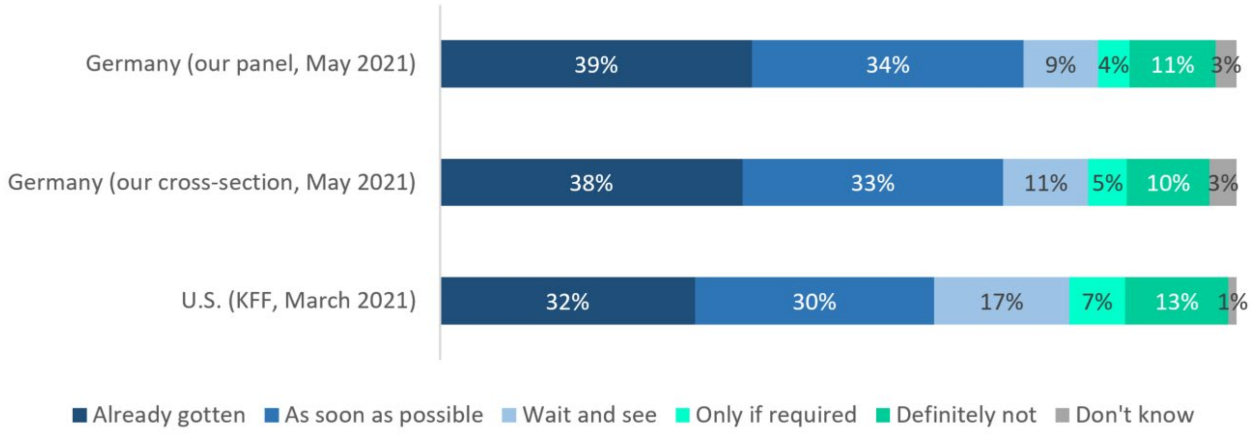

Fig. S6: Comparison of the German panel and the representative wave 3 cross-section and the German and U.S. responses to the Kaiser Family Foundaton questions. The figure compares our May survey results (both the entire sample in the May 2021 wave N=4,015, and those in the May 2021 wave that were also in the previous two waves, N=2,016) with the Kaiser Family Foundation's survey conducted between 15th and 22nd March, 2021 when the fraction of survey respondents already vaccinated is as nearly similar to our May 2021 German survey as possible. We selected this KFF survey because the distibution of vaccination attitudes among those remaining unvaccinated depends on the fraction already vaccinated (all of whom must have been willing). By the second half of April when the next KFF survey was conducted, the fraction already vaccinated had risen to 56 percent. We could not ask the KFF questions in the two earlier waves of our panel, because the KFF survey started only in December 2020. The KFF data are available under reference (4).

Recalculating our main results using separate samples of older East and West Germans

Below we compare the East and West German older cohorts (i.e., born before 1970, who had therefore reached adulthood before the end of the GDR) with respect to:

- the fraction who are consistently opposed to voluntary vaccination;
- the extent to which being consistently opposed is predicted by socio-demographic characteristics alone and by the full model including beliefs;
- and the fractions agreeing with and opposed to being vaccinated in at least one of the three waves.

We show that in all cases, results computed using the samples of older East and West Germans are very similar.

In Fig. S7, concerning our key finding, we show that the fraction consistently opposed to voluntary and enforced vaccination (responding 0 or 1 in all waves, orange slices) is very similar among older East and West Germans both if vaccinations are voluntary and enforced.

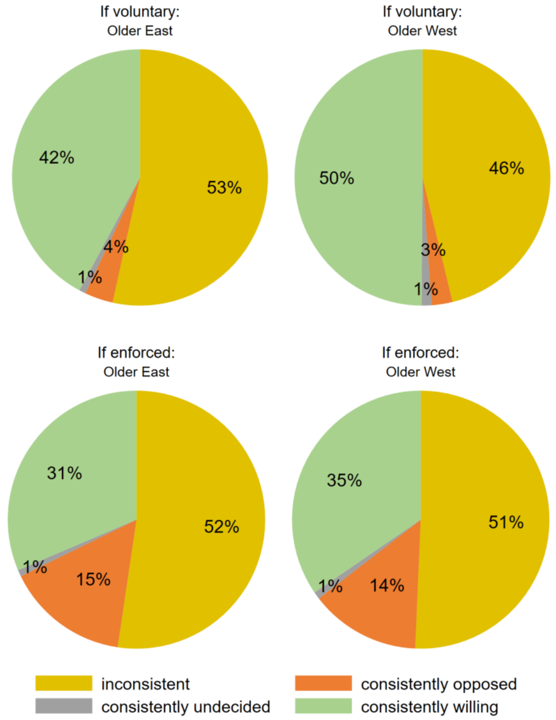

Fig. S7: **The distribution of consistently opposed (i.e., being opposed *in all 3 survey waves*), consistently willing, consistently undecided and inconsistent responses across the 3 waves for the voluntary and enforced cases among older East and West Germans.** Sample sizes: n=363 for older East Germans and n=874 for older West Germans.

Turning to our second key finding, Fig. S8 shows that for older East and West Germans alike, socioeconomic variables alone do not predict consistent opposition to voluntary vaccination (Tjur's R^2: East 0.015; West 0.009), while adding beliefs predicts substantial shares of the differences between those consistently opposed and others (East: 0.156; West: 0.212). A similar pattern holds for enforced vaccinations.

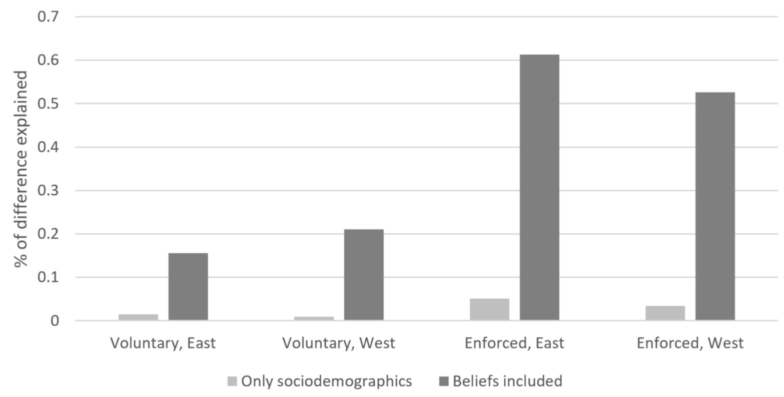

Fig. S8: **The fraction of difference between the consistently opposed and others explained by sociodemographic differences alone and the full model including beliefs (Tjur's R^2).** Sample sizes: n=357 for older East Germans and n= 851 for older West Germans.

Finally, we ask whether older East and West Germans differ in the fractions that were opposed to and agreed with being vaccinated, respectively, in at least one of our three waves. Again, East and West Germans do not differ in any appreciable way, as shown in Fig. S9.

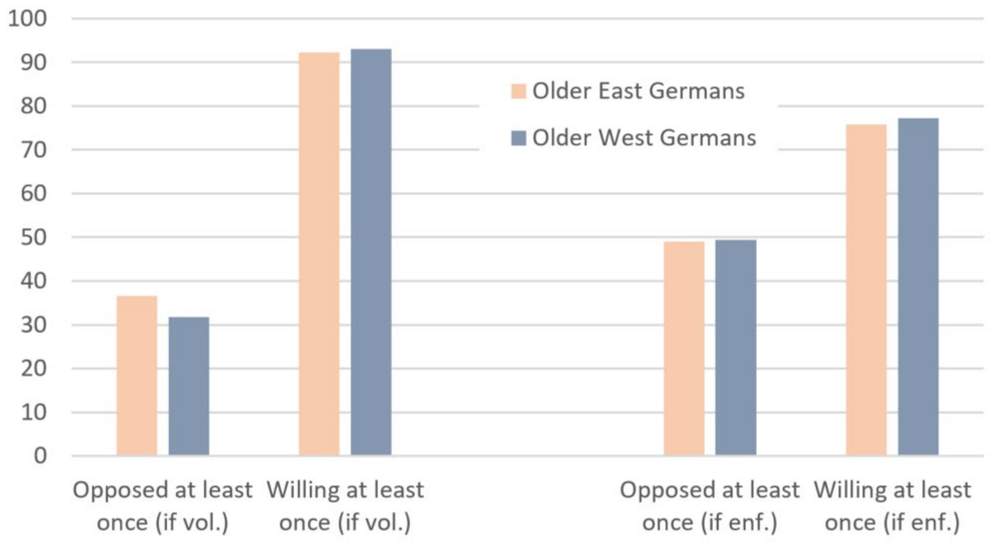

Fig. S9: Fractions of older East and West Germans who were opposed to and agreed with being vaccinated *in at least one wave of the panel*. Sample sizes: n=363 for older East Germans and n=874 for older West Germans.

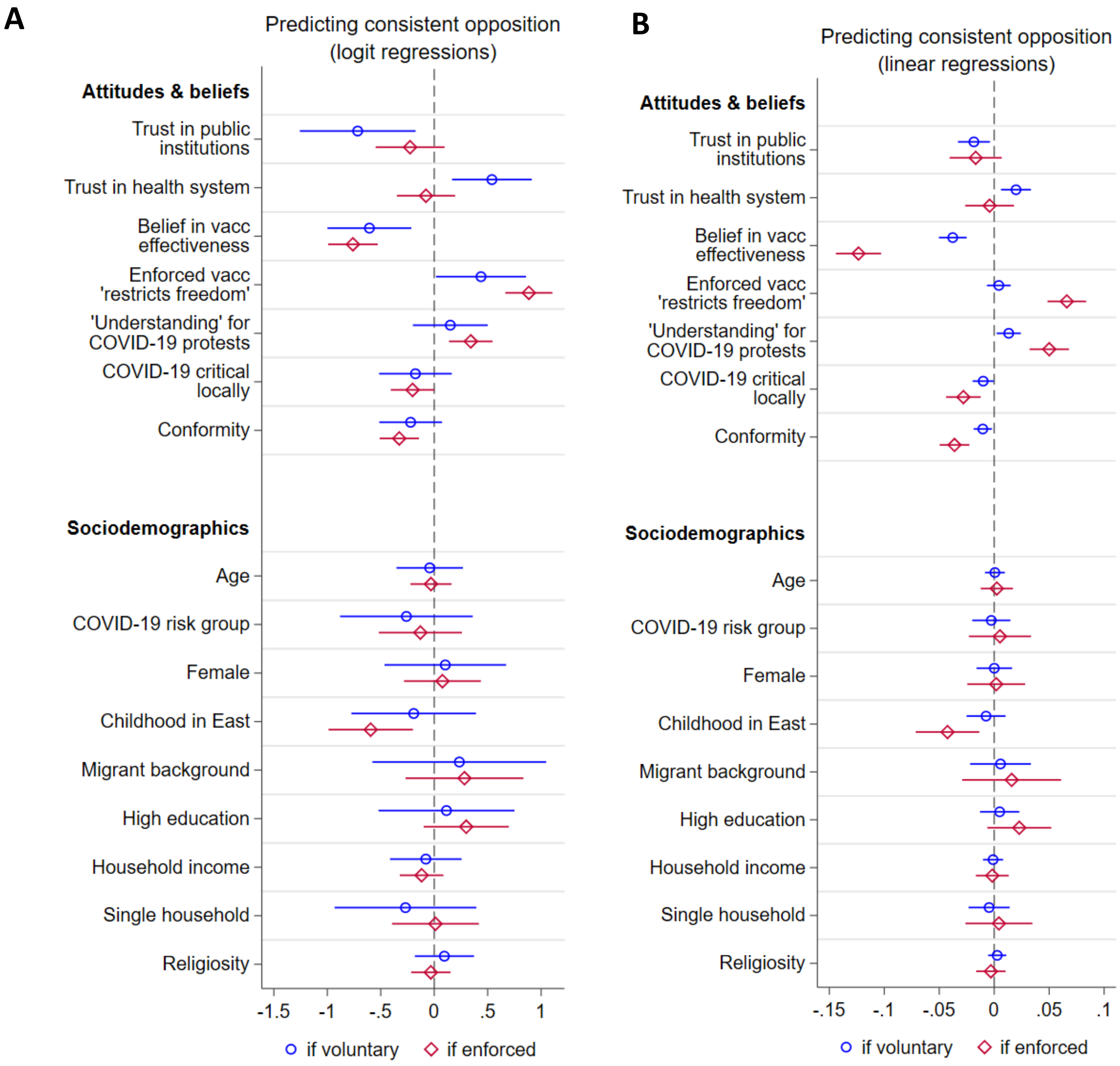

Fig. S10. Logit (A) and linear (B) regressions predicting consistent opposition to vaccination, that is responding 0 or 1 in all three survey waves. Shown are the estimated coefficients and the 95% CI's estimated with normalized variables (except for dummy variables). The beliefs variables are averaged across the survey waves (trust was asked in all three waves; beliefs in effectiveness, freedom restriction and understanding for protests in waves 2 and 3, and conformity only in wave 3). Tjur's goodness of fit measures are presented in Table S5.

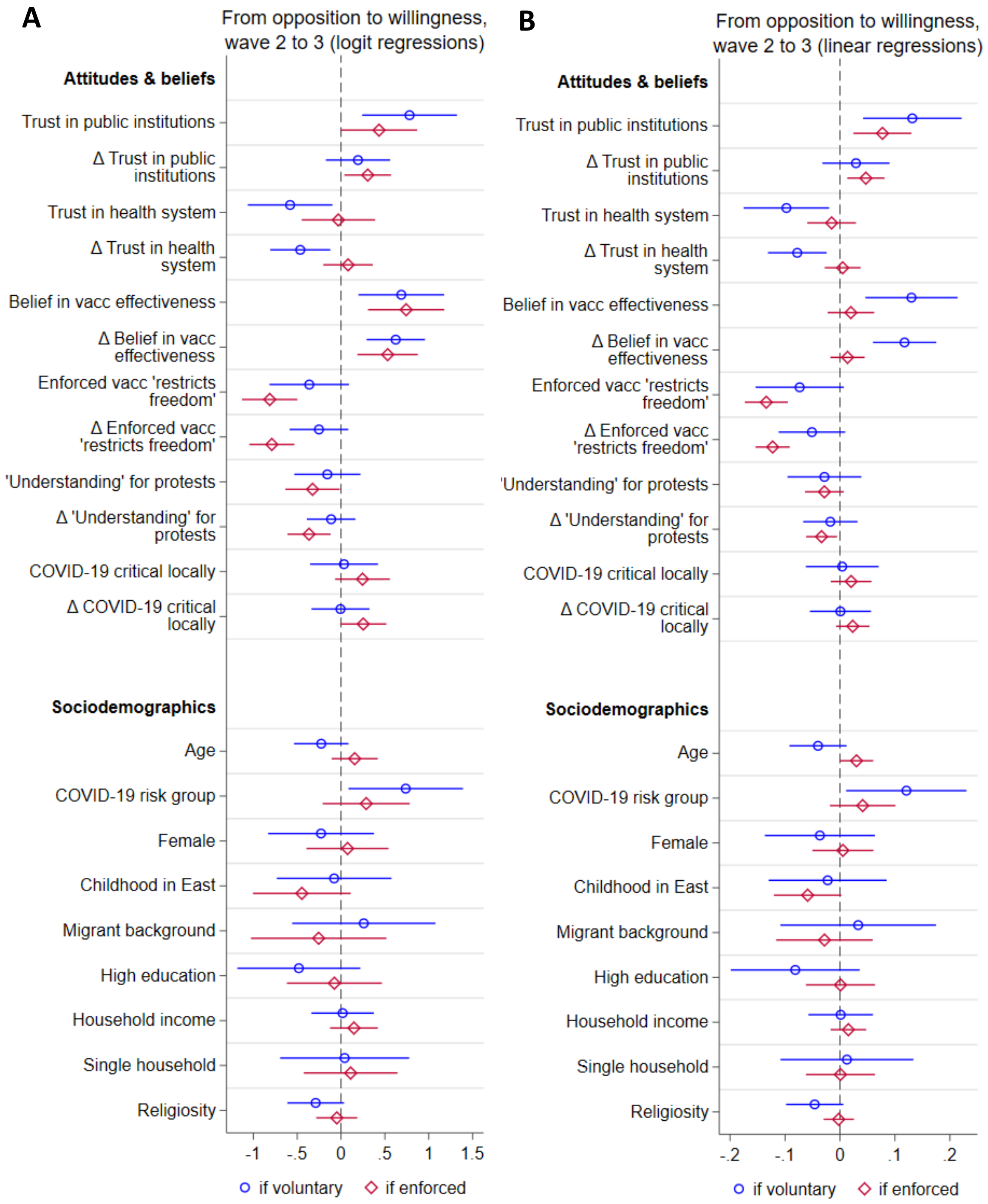

Fig. S11. Logit (A) and linear (B) regressions predicting those who switched from opposing vaccinations in wave 2 (levels 0 or 1) to supporting vaccinations in wave 3 (levels 3 or 4). Shown are the coefficients and 95% CI, estimated with normalized variables except for dummy variables. The level variables in attitudes and beliefs here all refer to wave 2. The Δ variables reflect the difference wave 3 - wave 2 and should be read as "increase in…". We could not perform the same analyzes for switching between waves 1 and 2 because we elicited the relevant attitudes and beliefs only from wave 2 on.

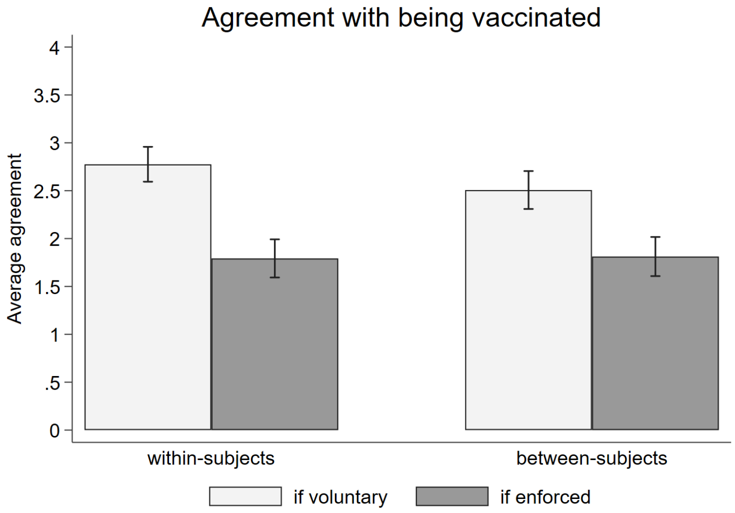

Fig. S12. Agreement with being vaccinated if voluntary and if enforced in within-subjects and between-subjects designs. In the between-subjects case in which there cannot be a demand effect, enforcement substantially reduces agreement. Thus, our evidence that a mandate induces a control averse response is not due to a demand effect of the within-subjects design. These treatments were implemented using a representative non-panel sample in wave 2 (within-subjects: n=245; between-subjects, voluntary: n=215; between-subjects, enforced: n=229).

Table S1. Number of participants in the three cross-section waves of the survey and the panel, dropouts and exclusion criteria. Exclusions according to the shown criteria were performed by the *surveyLab*, based on an independent quality check in which the authors of this paper were not involved.

The majority of those in the second row of the table were eliminated by the survey algorithm designed to ensure a representative sample (the appropriate shares of particular combinations of sociodemographics were required for representativeness, e.g., low educated young females from a given region).

	**Cross-section surveys**		
	**Wave 1**	**Wave 2**	**Wave 3**
Survey started	6,340	6,246	4,765
Respondent eliminated by representativeness algorithm (vast majority of cases) or survey not completed by respondent (fewer)	1,032	2,095	506
Very high number of missing answers	241	25	37
Nonsense responses to open questions	36	5	44
Speeders who completed the survey in less 40% of median interview duration	144	183	51
Straightlining (same responses across various question blocks)	88	45	100
**Cross-section sample size**	**4,799**	**3,893**	**4,027**
Respondents included in all 3 waves			2,044
Missing answer to at least one of the 6 questions on agreement with getting vaccinated (3 waves x 2 policies)			26
**Final panel sample size**			**2,018**

Table S2. Socio-demographics for the representative cross-sections and the panel waves. For birth year and agreement with being vaccinated, means (standard deviations) are shown. Double quota for East Germans were used. In the third wave, only those not "fully" vaccinated (i.e., not vaccinated twice) were asked the questions on agreement with being vaccinated.

	Wave 1		Wave 2		**Wave 3**	
Sociodemographics	Cross-section	Panel	Cross-section	Panel	**Cross-section**	**Panel**
N	4,799	2,018	3,893	2,018	**4,021**	**2,018**
Female	51%	47%	51%	47%	**49%**	**47%**
Year of birth	1972 (15.95)	1967 (14.00)	1971 (16.15)	1967 (14.00)	**1970 (16.22)**	**1967 (14.00)**
Education (low, middle, high)	31%, 33%, 36%	33%, 34%, 33%	34%, 32%, 34%	33%, 34%, 33%	**37%, 30%, 32%**	**33%, 34%, 33%**
Living in East Germany	32%	34%	33%	34%	**29%**	**34%**
COVID19-/Vaccination-related variables						
COVID-19 risk group	37%	41%	42%	46%	**43%**	**46%**
AfD voter (our survey)	11%	10%	10%	10%	**10%**	**9%**
AfD voter (Allensbach survey (5))	9%		10%		**10%**	
Agreement with being vaccinated if voluntary	2.86 (1.44)	2.87 (1.47)	2.80 (1.41)	2.86 (1.40)	**3.02 (1.33)**	**3.11 (1.31)**
Agreement with being vaccinated if enforced	2.39 (1.62)	2.54 (1.60)	1.92 (1.61)	2.01 (1.61)	**2.23 (1.63)**	**2.31 (1.64)**
Vaccination state:						
Received 1st shot					**31%**	**33%**
Vaccinated twice					**7%**	**6%**
Vaccination appointment					**8%**	**9%**
No appointment					**54%**	**52%**

Attitudes towards vaccination are very similar between the panel and the cross-section surveys, as are the vaccination status in the third wave, the level of education, and the fraction of East Germans. Support for the right-wing, anti-government party AfD is very similar in our panel and our cross-section surveys to others conducting regular surveys on political party preferences. The panel is somewhat older and more male than the cross-sections. But Figs. S10 and S11 show that neither age nor gender is an important predictor of our variables of interest. Similarly, Table S3 shows that using population sample weights on the panel gives similar estimates of opposition to vaccination.

Table S3. Comparing opposition to vaccination with and without sample weights in the panel sample.

	Unweighted	Sample weights
Consistent oppontents if voluntary	3.32%	3.57%
Average opposed across 3 waves if voluntary	16.90%	16.99%
Consistent oppontents if enforced	16.50%	17.21%
Average opposed across 3 waves if enforced	33.17%	34.48%

Table S4. Explaining the variables in the regressions. Note that in the regression models predicting consistent opposition (Fig. 3A in the paper and Fig. S10), we use the average of the beliefs and attitudes in the three survey waves (or the average of the second and third waves for questions which were not asked in the first wave). To predict moving from opposition to vaccination in wave 2 towards willingness in wave 3 (Fig. 3B in the paper and Fig. S11), we use the wave 2 beliefs and attitudes as the level variables. Therefore, we indicate the distributions for both cases. Q1 (Q2 and Q3) refer to the 1st quartile (median and 3rd quartile) of the distributions. In all regressions, sociodemographic variables which are dummies were used from wave 2, and continuous variables are averaged over the 3 survey waves (e.g., income).

Variable	Description and remarks	Survey question	Distribution
Trust in public institutions	Average of 4 measures of trust in public institutions: trust in federal government, trust in state government, trust in science, and trust in media.<br><br>The 4 measures are highly correlated (mean correlation of the 3 waves average = 0.70, min = 0.62). In a PCA, the first component explains 78% of the variance, suggesting one underlying component.	Here you can now see a number of public bodies and institutions. How much confidence do you generally have in them?<br>Federal government<br>State government<br>Experts from science<br>Media<br>(7-point Likert scale ranging from 1 “no confidence at all” to 7 “a great deal of confidence”) (6)	*Average across the 3 waves:*<br>Mean=4.06, SD=1.43, Q1=3.08, Q2=4.25, Q3=5.17, missing: n=0.<br><br>*Wave 2:*<br>Mean=4.18, SD=1.56, Q1=3.25, Q2=4.25, Q3=5.5, missing: n=0.
Δ Trust in public institutions	Difference in *Trust in public institutions* between wave 3 and wave 2 (wave 3 – wave 2).		Mean= -0.34, SD=0.88, Q1=-0.75, Q2= -0.25, Q3=0.25, missing: n=1.
Trust in health system		Here you can now see a number of public bodies and institutions. How much confidence do you generally have in them?<br>Health system<br>(7-point Likert scale ranging from 1 “no confidence at all” to 7 “a great deal of confidence”) (6)	*Average across the 3 waves:*<br>Mean=4.73, SD=1.43, Q1=4, Q2=5, Q3=5.67, missing: n=0.<br><br>*Wave 2:*<br>Mean=4.82, SD=1.64, Q1=4, Q2=5, Q3=6, missing: n=3.
Δ Trust in health system	Difference in *Trust in health system* between wave 3 and wave 2 (wave 3 – wave 2).		Mean= -0.25, SD=1.24, Q1= -1, Q2=0, Q3=0, missing: n=6.
Belief in vacc effectiveness	Survey question included only in waves 2 and 3.	What do you think: How effective are the following measures (if most people comply) in containing the spread of the coronavirus?<br>… vaccination against the coronavirus (5-point Likert scale ranging from 0 “not at all effective” to 4 “highly effective”)	*Average across waves 2 and 3:*<br>Mean=3.05, SD=1.11, Q1=2.5, Q2=3.5, Q3=4, missing: n=0.<br><br>*Wave 2:*<br>Mean=2.82, SD=1.30, Q1=2, Q2=3, Q3=4, missing: n=2.

Δ Belief in vacc effectiveness	Difference in *Belief in vacc effectiveness* between wave 3 and wave 2 (wave 3 – wave 2).		Mean= 0.46, SD=1.05, Q1=0, Q2=0, Q3=1, missing: n=7.
Enforced vacc 'restricts freedom'	Survey question included only in waves 2 and 3.<br><br>This question was not asked of the respondents in wave 3 who were already vaccinated twice.	Assume that the following anti-COVID-19 measures are mandatory and checked.<br>To what extent do you feel this restricts your freedom?<br>… vaccination against the coronavirus (5-point Likert scale ranging from 0 "not at all restricted in my freedom" to 4 "absolutely restricted in my freedom")	*Average across waves 2 and 3:* Mean=1.51, SD=1.43, Q1=0, Q2=1, Q3=2.5, missing: n=0.<br><br>*Wave 2:* Mean=1.57, SD=1.61, Q1=0, Q2=1, Q3=3, missing: n=6.
Δ Enforced vacc 'restricts freedom'	Difference in *Enforced vacc 'restricts freedom'* between wave 3 and wave 2 (wave 3 – wave 2).<br><br>This difference of course cannot be computed for those vaccinated twice in wave 3 (see above).		Mean= -0.13, SD=1.55, Q1= -1, Q2=0, Q3=0, missing: n=138.
'Understanding' for protests	Survey question included only in waves 2 and 3.	For several months now, people in Germany have been demonstrating at so-called Corona protests or *Querdenker* ("lateral thinker") protests against the policies and the actions of the federal and state governments. Some people are critical of these demonstrations, while others find them justified.<br>Do you have any understanding for these demonstrations?<br>(no, I have no understanding (coded 1) - little understanding - undecided - some understanding - yes, fully completely (coded 5))	*Average across waves 2 and 3:* Mean=2.05, SD=1.25, Q1=1, Q2=1.5, Q3=3, missing: n=0.<br><br>*Wave 2:* Mean=2.00, SD=1.31, Q1=1, Q2=1, Q3=3, missing: n=3.
Δ 'Understanding' for protests	Difference in *'Understanding' for protests* between wave 3 and wave 2 (wave 3 – wave 2).		Mean= 0.09, SD=0.94, Q1=0, Q2=0, Q3=0, missing: n=12.
COVID-19 critical locally	Survey question included only in waves 2 and 3.	How critical do you think the COVID-19 situation currently is in your region? (9-point Likert scale ranging from 1 "not critical at all" to 9 "highly critical")	*Average across waves 2 and 3:* Mean=5.42, SD=1.94, Q1=4, Q2=5.5, Q3=7, missing: n=0.<br><br>*Wave 2:* Mean=5.74, SD=2.24, Q1=4, Q2=6, Q3=7, missing: n=4.
Δ COVID-19 critical locally	Difference in *COVID-19 critical locally* between wave 3 and wave 2 (wave 3 – wave 2).		Mean= -0.65, SD=2.03, Q1=-2, Q2=-1, Q3=0, missing: n=10.

Conformity	Survey questions included only in wave 3.	Average score on Mehrabian & Stefl's conformity scale (7) consisting of 11 items, ranging from 0 to 6.	Mean=2.08, SD=0.84, Q1=1.45, Q2=2.09, Q3=2.64, missing: n=0.
Age	In years at 2020, the time of the panel start, computed from year of birth.		Mean=53.31 years, SD=14.00, Q1=43, Q2=55, Q3=64.
COVID-19 risk group	Dummy indicating whether a person would be at high risk when infected with COVID-19.	Do you belong to the COVID-19 risk group due to pre-existing health issues? (yes/no)	*Wave 2:* 46% risk group, 53% not risk group, 1% missing (n=17).
Female	Dummy variable which takes the value one for female and zero for male.		47% female, 53% male.
Childhood in East	Dummy variable taking the value one if a person spent her or his childhood in a federal state of East Germany. It takes the value zero for West Germans and is missing otherwise.	In which federal state did you spend most of your childhood?	30% East, 69% West, 1% missing (n=21).
Migrant background	Dummy variable taking the value one if the respondent and/or at least one of the respondent's parents were not born in Germany.		*Wave 2:* 9.4% migrants, 90.2% non-migrants, 0.4% missing (n=8).
High education	Dummy for high levels of schooling. It takes the value zero for low and intermediate levels of schooling.	What is your highest school leaving certificate? (subjects choose from a list of options)	*Wave 2:* 33% high education, 67% middle or low education.
Household income	Subjects could choose to answer an open or a categorical question on net household income (in euros). The variable is constructed by combining the answers to both questions (done by the surveyLab).	6 categories where 1 refers to <900 euros and 6 refers to >=6000 euros.	*Average across the 3 waves:* Mean=3.22, SD=1.19, Q1=2, Q2=3, Q3=4, missing: n=13.
Single household	Dummy which indicates whether the person lives alone. It takes the value zero if more than one person live in the respondent's household.	How many people currently live in your household, including yourself?	*Wave 2:* 33% single households, n=0 missing (answer was mandatory).
Religiosity	Continuous measure	One can also be religious without belonging to a religious community. Please imagine a scale from 0 to 10. 0 means that you are "not religious at all", 10 means that you are "very religious". Where would you place yourself? (11-point Likert scale)	*Average across the 3 waves:* Mean=2.94, SD=2.99, Q1=0, Q2=2, Q3=5.67, missing: n=0.

Predicting consistent opposition and movement out of opposition to vaccinations.

In Table S5 and Figs. S10 and S11 we present information underlying Fig. 3 of the paper and the passage surrounding it. We estimate both logit and linear probability models (the former presented in the paper). Because the mean of the dependent variable is close to zero for predicting consistent opposition (very few consistently opposed), a large fraction of the predicted values lie outside the unit interval when we implement the linear probability model, so that, following the evidence in Horace and Oaxaca (8), we use the logit to present our main findings and complement them with linear probability models for a more intuitive interpretation of the coefficients.

Table S5 provides evidence that for both the logit and linear probability models socio-demographic variables alone (including the indirect effects of their covariation with beliefs and attitudes) account for far less of the differences than the full model in which attitudes and beliefs are included.

Table S5. Goodness of fit predicting consistent opposition to vaccination and movement from opposition to agreement between waves 2 and 3. The binary dependent variable is 1 for those opposed to vaccination in all three waves (and 0 for all others) in the top panel, and 1 for those who were opposed in wave 2 but agreed with being vaccinated in wave 3 in the bottom panel (and 0 for all others). The last column gives the number of 1's in the dependent variable (DV) in each regression. In the bottom panel the column headed N gives total number opposed in wave 2, which is the basis for the regressions. For the linear probability model, Tjur's R^2 is identical with the conventional R^2 for the estimated equation.

Model				Tjur's R^2	Pseudo R^2	N	N (DV=1)
Predicting persistent opposition to vaccination	Logit models	Only socio-demographics	voluntary	0.0075	0.0244		67
			enforced	0.0309	0.0349		326
		Attitudes & beliefs incl.	voluntary	0.1532	0.2731		67
			enforced	0.4829	0.4685	1,959	326
	Linear models	Only socio-demographics	voluntary	0.0073			67
			enforced	0.0309			326
		Attitudes & beliefs incl.	voluntary	0.1071			67
			enforced	0.4365			326
Predicting from opposition in wave 2 to agreement in wave 3	Logit models	Only socio-demographics	voluntary	0.0733	0.0558	362	209
			enforced	0.0530	0.0488	755	174
		Attitudes & beliefs incl.	voluntary	0.3542	0.2887	325	174
			enforced	0.3644	0.3517	741	172
	Linear models	Only socio-demographics	voluntary	0.0738		362	209
			enforced	0.0510		755	174
		Attitudes & beliefs incl.	voluntary	0.3480		325	174
			enforced	0.3097		741	172

In Fig. S10 (predicting consistent opposition to vaccinations) the only socio-demographic variable of any importance is (for the case of enforced vaccinations) the dummy variable indicating that the respondent spent their childhood in the territory of the former GDR. This is consistent with the fact that East Germans, especially those raised under Communist Party rule, have been found to be less control averse (9, 10). In Fig. S11 (predicting switching from opposed to favoring vaccinations) this holds to a lesser extent. Nonetheless, Tjur's R^2 in Table S5 shows that sociodemographic measures, taken as a whole, have very little explanatory power.

Transition matrices and stationary distributions

Figures 2 and 3 in the paper give an indication of the persistent or transient nature of vaccination attitudes. A more complete presentation of our evidence on these dynamics is afforded by the transition matrices P^{12} and P^{23} recording changes in attitudes between the first and second, as well as the second and third waves of the survey respectively. The corresponding elements are $p_{ij}^{t,t+1}$, the probability that an individual responding with Likert scale score *i* in the earlier wave *t* responds with a score *j* in the later wave *t*+1 where $i, j \in [0,1,2,3,4]$. We present the wave 1 to wave 2 and wave 2 to wave 3 *P* matrices for the cases of mandated and voluntary vaccination in Table S6.

These transition matrixes allow a different perspective on the question of transience, namely the stationary (or long run equilibrium) distribution of attitudes in the population of the underlying Markov process over the five Likert score states for an unchanging irreducible *P* matrix. Where $\lambda_k \text{ for } k \in [0,1,2,3,4]$ is the fraction of the population expressing each of the five vaccination attitudes, the following vector represents a distribution of the population: $\lambda = [\lambda_0 \; \lambda_1 \; \lambda_2 \; \lambda_3 \; \lambda_4]$. The stationary distribution λ^* is defined by $\lambda^* P = \lambda^*$, that is, the λ that solves:

$$[\lambda_0 \quad \lambda_1 \quad \lambda_2 \quad \lambda_3 \quad \lambda_4] \begin{bmatrix} p_{00} & p_{01} & .. & .. & p_{04} \\ p_{10} & & & & \\ .. & & p_{02} & & .. \\ .. & & & & \\ p_{40} & .. & .. & .. & p_{44} \end{bmatrix} = [\lambda_0 \quad \lambda_1 \quad \lambda_2 \quad \lambda_3 \quad \lambda_4]$$

and

$$\lambda_0 + \lambda_1 + \lambda_2 + \lambda_3 + \lambda_4 = 1$$

To be clear, the stationary distribution is not a measure of consistent opposition in the sense we have described it in the paper, namely the extent to which individuals express unchanging vaccination attitudes over time (these are the diagonal elements in the *P* matrix, p_{ii}). Instead, the *λ* vector is the distribution that would persist in the long run were *P* not to change, with those individuals making up the five fractions of the population changing from period to period, the distribution itself persisting but not the membership of its states.

Table S6 provides additional information on the transience of opposition. Panel A shows, for example, that of those strongly disagreeing with being vaccinated voluntarily in wave 1, only 46 percent (that is 37 percent responding 0 plus 9 percent responding 1) disagreed (either weakly or strongly) in wave 2. The stationary distributions appear in bold below the *P* matrices in Table S6.

Table S7 presents reduced transition matrices and associated stationary distributions with just three states: vaccine willingness, vaccine opposition and undecided. We can see that opposition to voluntary vaccination in the stationary distribution is somewhat less in the wave 2 to wave 3 *P* matrix than in the wave 1 to wave 2 *P* matrix (11 percent rather than 18 percent). For the case of enforced vaccinations, there is a very substantial drop in the weight of vaccine opposition in the stationary distribution, from over half based on the first and second waves to a quarter of the sample based on the second and third waves. Panel E includes the 7 percent who were vaccinated twice in the 'willing' category for the case of voluntary vaccination and shows that the inclusion of those vaccinated twice does not qualitatively change the conclusions one would draw from the transition matrix and the associated stationary distribution.

Table S6. Transition matrices and stationary distributions for the full 5-point Likert scores. The transition matrices including the third wave (panels C, D, E and F) are based on the 93 percent of the sample that was not vaccinated twice in May 2021.

**A. If voluntary**	Agreement in wave 2					
Agreement in wave 1	0	1	2	3	4	Total
0	0.37	0.09	0.12	0.07	0.35	1
1	0.17	0.12	0.24	0.17	0.29	1
2	0.10	0.08	0.27	0.24	0.31	1
3	0.06	0.08	0.17	0.33	0.36	1
4	0.07	0.04	0.12	0.14	0.63	1
**Stationary distribution**	**0.11**	**0.07**	**0.16**	**0.19**	**0.47**	**1**

**B. If enforced**	Agreement in wave 2					
Agreement in wave 1	0	1	2	3	4	Total
0	0.79	0.06	0.08	0.04	0.04	1
1	0.31	0.21	0.21	0.15	0.12	1
2	0.26	0.20	0.26	0.16	0.13	1
3	0.14	0.11	0.23	0.29	0.23	1
4	0.12	0.09	0.15	0.14	0.49	1
**Stationary distribution**	**0.49**	**0.11**	**0.15**	**0.11**	**0.15**	**1**

**C. If voluntary**	Agreement in wave 3					
Agreement in wave 2	0	1	2	3	4	Total
0	0.38	0.05	0.09	0.09	0.39	1
1	0.10	0.10	0.15	0.17	0.47	1
2	0.07	0.05	0.22	0.20	0.46	1
3	0.03	0.04	0.19	0.26	0.48	1
4	0.06	0.02	0.07	0.10	0.75	1
**Stationary distribution**	**0.08**	**0.03**	**0.11**	**0.13**	**0.64**	**1**

**D. If enforced**	Agreement in wave 3					
Agreement in wave 2	0	1	2	3	4	Total
0	0.64	0.09	0.10	0.04	0.13	1
1	0.19	0.14	0.26	0.18	0.24	1
2	0.10	0.09	0.25	0.20	0.36	1
3	0.07	0.09	0.20	0.24	0.40	1
4	0.05	0.03	0.09	0.09	0.74	1
**Stationary distribution**	**0.17**	**0.06**	**0.13**	**0.12**	**0.51**	**1**

**E. If voluntary**	Agreement in wave 3					
Agreement in wave 1	0	1	2	3	4	Total
0	0.25	0.07	0.13	0.12	0.42	1
1	0.11	0.06	0.16	0.16	0.51	1
2	0.11	0.07	0.27	0.18	0.38	1
3	0.04	0.02	0.14	0.28	0.51	1
4	0.06	0.02	0.08	0.10	0.73	1

**F. If enforced**	Agreement in wave 3					
Agreement in wave 1	0	1	2	3	4	Total
0	0.68	0.09	0.13	0.03	0.08	1
1	0.31	0.17	0.16	0.16	0.19	1
2	0.21	0.11	0.25	0.16	0.27	1
3	0.14	0.09	0.22	0.22	0.33	1
4	0.08	0.05	0.11	0.13	0.63	1

Table S7. Transition matrices and stationary distributions for a 3-state Markov process (opposed, undecided, and willing). This simpler transition matrix treats as single states *opposition* (either strong or weak, Likert scale answers 0 or 1) and *willingness* (either weak or strong, Likert scale answers 3 or 4) with *undecided* as the third, intermediate state (Likert scale answer 2). As done for Table S6, the matrices including the third wave (panels C, D, F and G) include the 93 percent that were not vaccinated twice in May 2021. Panels E and H include the 7 percent who were vaccinated twice in the 'willing' category for the case of voluntary vaccination and show that the inclusion of those vaccinated twice does not qualitatively change the conclusions one would draw from the transition matrix and the associated stationary distribution. Transience is also evident from the self-reported behavioral data: Among those opposed to voluntary vaccination in wave 2, 38.5 percent were vaccinated at least once or had an appointment in wave 3. Among the undecided in wave 2, 44.6 percent were vaccinated at least once or had an appointment in wave 3, and among those willing if vaccinations are voluntary in wave 2, 51.0 percent were vaccinated at least once or had an appointment in wave 3 (remember that vaccine availability as well as appointments were very limited at the time of our survey wave 3).

A. If voluntary (all)	Response in wave 2			
Response in wave 1	opposed	undecided	willing	Total
opposed	0.41	0.16	0.43	1
undecided	0.18	0.27	0.55	1
willing	0.12	0.13	0.76	1
**Stationary distribution**	**0.18**	**0.16**	**0.67**	**1**

B. If enforced (all)	Response in wave 2			
Response in wave 1	opposed	undecided	willing	Total
opposed	0.77	0.11	0.12	1
undecided	0.45	0.26	0.29	1
willing	0.22	0.17	0.61	1
**Stationary distribution**	**0.56**	**0.15**	**0.29**	**1**

C. If voluntary (93%)	Response in wave 3			
Response in wave 2	opposed	undecided	willing	Total
opposed	0.35	0.11	0.54	1
undecided	0.12	0.22	0.66	1
willing	0.08	0.11	0.82	1
**Stationary distribution**	**0.11**	**0.12**	**0.77**	**1**

D. If enforced (93%)	Response in wave 3			
Response in wave 2	opposed	undecided	willing	Total
opposed	0.62	0.14	0.24	1
undecided	0.20	0.25	0.56	1
willing	0.11	0.12	0.77	1
**Stationary distribution**	**0.25**	**0.15**	**0.60**	**1**

E. If voluntary (all)	Response in wave 3			
Response in wave 2	opposed	undecided	willing	Total
opposed	0.32	0.10	0.58	1
undecided	0.12	0.21	0.67	1
willing	0.07	0.10	0.83	1
**Stationary distribution**	**0.10**	**0.11**	**0.79**	**1**

F. If voluntary (93%)	Response in wave 3			
Response in wave 1	opposed	undecided	willing	Total
opposed	0.28	0.14	0.58	1
undecided	0.17	0.27	0.56	1
willing	0.08	0.09	0.83	1

G. If enforced (93%)	Response in wave 3			
Response in wave 1	opposed	undecided	willing	Total
opposed	0.70	0.14	0.16	1
undecided	0.32	0.25	0.43	1
willing	0.15	0.14	0.71	1

H. If voluntary (all)	Response in wave 3			
Response in wave 1	opposed	undecided	willing	Total
opposed	0.27	0.13	0.60	1
undecided	0.16	0.25	0.58	1
willing	0.07	0.09	0.84	1

References

1. Worldometer. *Worldometers coronavirus*, https://www.worldometers.info/coronavirus/country/germany/ (2022), accessed: February 8, 2022.
2. E. Mathieu *et al.*, A global database of COVID-19 vaccinations. *Nature Human Behaviour* **5**, 947-953 (2021).
3. J. Rudnicka. *Kinder und Jugendliche in Deutschland nach Altersgruppen 2020*, https://de.statista.com/statistik/daten/studie/1174053/umfrage/minderjaehrige-in-deutschland-nach-altersgruppen/ (2021), accessed: 5 August 2021.
4. L. Hamel, L. Lopes, G. Sparks, A. Kirzinger, A. Kearney, M. Stokes, M. Brodie. *KFF COVID-19 Vaccine Monitor: January 2022*, https://www.kff.org/coronavirus-covid-19/poll-finding/kff-covid-19-vaccine-monitor-january-2022/ (2022), accessed: February 8, 2022.
5. Allensbach Institut für Demoskopie. *Wenn am nächsten Sonntag Bundestagswahl wäre...* https://www.wahlrecht.de/umfragen/allensbach.htm (2021), accessed: 22 September 2021.
6. R. Inglehart, C. Haerpfer, A. Moreno, C. Welzel, K. Kizilova, J. Diez-Medrano, M. Lagos, P. Norris, E. Ponarin & B. Puranen et al. (eds.). *World Values Survey: Round Six*, http://www.worldvaluessurvey.org/WVSDocumentationWV6.jsp (2014), accessed: June 15, 2020.
7. A. Mehrabian, C. A. Stefl, Basic temperament components of loneliness, shyness, and conformity. *Social Behavior and Personality* **23**, 253-263 (1995).
8. W. C. Horrace, R. L. Oaxaca, Results on the bias and inconsistency of ordinary least squares for the linear probability model. *Economics Letters* **90**, 321-327 (2006).
9. K. Schmelz, A. Ziegelmeyer, State Coercion and Control Aversion: Evidence from an Internet Study in East and West Germany. https://ideas.repec.org/p/twi/respas/0117.html (February 2020).
10. K. Schmelz, Enforcement may crowd out voluntary support for COVID-19 policies, especially where trust in government is weak and in a liberal society. *Proceedings of the National Academy of Sciences of the United States of America (PNAS)* **118** (2021).
